# Molecular Docking and Multivariate Analysis of Xanthones as Antimicrobial and Antiviral Agents

**DOI:** 10.3390/molecules200713165

**Published:** 2015-07-21

**Authors:** Freddy A. Bernal, Ericsson Coy-Barrera

**Affiliations:** Laboratorio de Química Bioorgánica, Departamento de Química, Facultad de Ciencias Básicas y Aplicadas, Universidad Militar Nueva Granada, Cundinamarca 250240, AA 49300, Colombia; E-Mail: freddy.bernal@unimilitar.edu.co

**Keywords:** target enzymes, antifungal, antiviral, xanthones, molecular docking, multivariate analysis

## Abstract

Xanthones are secondary metabolites which have drawn considerable interest over the last decades due to their antimicrobial properties, among others. A great number of this kind of compounds has been therefore reported, but there is a limited amount of studies on screening for biological activity. Thus, as part of our research on antimicrobial agents of natural origin, a set of 272 xanthones were submitted to molecular docking (MD) calculations with a group of seven fungal and two viral enzymes. The results indicated that prenylated xanthones are important hits for inhibition of the analyzed enzymes. The MD scores were also analyzed by multivariate statistics. Important structural details were found to be crucial for the inhibition of the tested enzymes by the xanthones. In addition, the classification of active xanthones can be achieved by statistical analysis on molecular docking scores by an affinity-antifungal activity relationship approach. The obtained results therefore are a suitable starting point for the development of antifungal and antiviral agents based on xanthones.

## 1. Introduction

Despite great efforts to eradicate different human diseases, many of them remain as current health problems. Of these, infectious diseases represent one of the most important challenges due to the very high levels of morbidity and mortality they cause [1]. The most common medical treatments in infectious disease management consist of chemotherapeutics based on typical antibiotic drugs, but decreased efficacy along the time has been demonstrated. The failures of chemotherapy have been rationalized by three reasons: development of resistance, new emerging infections and re-emerging diseases [1]. Therefore, permanent drug discovery and research programs are still required. In depth, several research groups are developing ongoing studies on new antimicrobial agents around the world. Resistance development has been described as a natural evolutionary mechanism in the microorganisms, but it has also been attributed to an undesirable side-effect of the use of antibiotics even at sub-lethal concentrations [2]. Hence, in addition to hit and lead searching for structures, new therapeutic targets should be found in order to overcome resistance problems. Recently, new target searching has been proposed as a suitable strategy in anti-infective drug discovery [3].

On the other hand, appropriate screening methods for finding lead structures are highly required. In this regard, high-throughput screening (HTS) methods have become an important tool for pharmaceutical companies. Nevertheless, HTS is typically costly and time-consuming; it requires the development of specific assays for selected pharmaceutical targets and a compound library must firstly be synthesized [4,5]. Therefore, virtual screening (VS) methods have been developed and they have demonstrated remarkable advantages over HTS, including fast and cheap identification of lead and hit structures [4]. VS has even been recently reported as a key tool for drug repositioning [6]. The 3D structure-based VS approach, known as molecular docking [7], has become one of the most important drug discovery methods in recent years. Molecular docking comprises two main steps [8]: searching and scoring. Through the first one, a thermodynamic modeling to multiple ligand-protein poses is explored in order to search for local minima. By the last one, quality of the found docking poses is evaluated, so that some classification of the tested ligands can be achieved [8]. Among the advantages of molecular docking, the possibility to analyze new unproved therapeutic targets can be emphasized, but in order to get correct and validated prediction of the interactions between the ligand and the enzyme, search algorithms and scoring functions as well as adequate protocols must be permanently reviewed and improved [9,10].

Since traditional antibiotics are mainly natural product and natural product-derived compounds, and the natural compounds are still considered an important starting point in screening processes [1,11], drug discovery programs based on those have been carried out [12]. From the vast number of reported natural compounds, xanthones represent a great class of secondary metabolites with a limited amount of reported screening studies. Xanthones occur in some higher plant families, lichen and fungi [13]. Their distribution in plant families is restricted, so that they are considered as chemomarkers for such species. In spite of their limited distribution, numerous natural xanthones have been reported [13]. Several studies on the biological activity of xanthones have been described, including hypoglycemic, DNA polymerase inhibitor, anti-inflammatory, and antiviral activities [13]. Moreover, the anti-infective potential of several xanthones has also been stated [14,15,16,17,18,19,20,21,22,23].

One of the most detrimental viral infections is that due to HIV, therefore, the development of anti-HIV/AIDS drugs has conducted and a great number of such drugs have emerged in the last decades [24]. All commercially available antiviral drugs against HIV are reverse transcriptase (RT) inhibitors and/or protease inhibitors [24]. RT is an HIV enzyme which is responsible for converting the viral RNA to double-stranded DNA in the cytoplasm of infected cells [25,26]. Hence, RT has been considered as crucial target in antiviral drug discovery programs, including computer-based approaches. Other important target in antiviral drug development is GP120 [27], a surface glycoprotein essential for viral infection [27]. Moreover, GP120 has also been identified as a facilitator of viral persistence by influencing the T cell immune response of the host [28]. RT and GP120 crystal structures have been well determined and molecular docking studies looking for new lead structures against HIV are thus feasible.

Common targets in antimicrobial drug therapy are ergosterol synthesis and cell wall β-1,3-glucan synthesis [29,30]. Enzymes responsible for the cell walls have been not crystalized, ergo molecular docking studies on such targets are not possible. On the other hand, ergosterol contributes to structural functions in fungi and bacteria, as well as to several important functions, including membrane integrity and signaling [31,32]. Oxidative removal of the 14α-methyl group from sterol precursors after cyclization of squalene 2,3-epoxide affords ergosterol by action of sterol 14α-demethylase [33]. In depth, inhibition of sterol 14α-demethylase enzyme results in a lethal process for unicellular organisms [33]. Besides the abovementioned targets, other enzymes important for vital fungi processes can be proposed in antifungal drug discovery programs. Therefore, we selected and analyzed as new therapeutic antifungal targets another six fungal enzymes: ribonuclease F1, α-fucosidase, nitric oxide reductase, *N*-myristoyltransferase, trichodiene synthase and α-L-arabinofuranosidase. These enzymes were used in the present study due to their important roles in the microbial metabolism as follows: ribonucleases play important roles in several fungi pathways and ribonuclease F1 catalyzes the hydrolysis of the phosphodiester bond at the 3′-side of guanosine in single-stranded RNA [34]. Fucosylated glycans play important roles in several pathological processes, including adhesion processes of pathogens [35], which led us to identify α-fucosidase as a possible drug target. On the other hand, as a detoxification mechanism some pathogens are able to transform NO into N_2_O by the action of nitric oxide reductase. NO can be generated as a defense response in the host during an infection [36]. For its part, *N*-myristoyl transferase catalyzes the transfer of myristate to the *N*-terminal glycine residue of a variety of eukaryotic cellular and viral proteins [37]. This enzyme is involved in signaling and plays an essential role in the growth of human pathogens [38]. Finally, trichodiene synthase catalyzes the cyclization of farnesyl pyrophosphate to afford trichodiene, a hydrocarbon involved as an intermediate in the synthesis of several mycotoxins [39]. Thereof, a set of 272 xanthones were randomly selected from literature reports [15,16,17,20,40,41] and submitted to molecular docking against the enzymatic pool above mentioned, which consist of seven fungal and two viral important proteins. The aim of the present paper was to establish possible new therapeutic targets and lead structures for the treatment of fungal and/or viral infections as well as to determine the structure—affinity relationship by means of multivariate analysis.

## 2. Results and Discussion

### 2.1. Affinity Energy Trends and Multivariate Statistical Analysis

**Figure 1:**
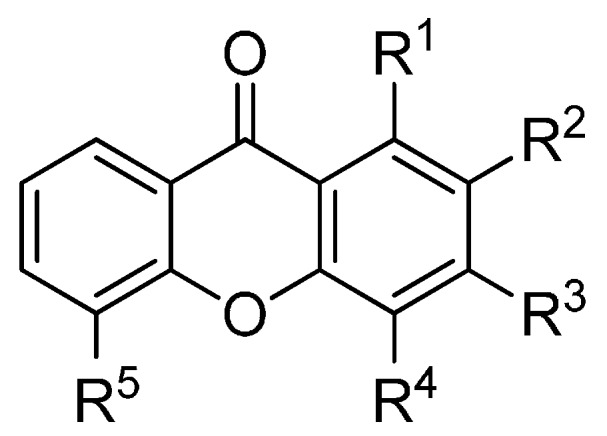
Chemical structures of tested monooxygenated xanthones.

**Figure 2:**
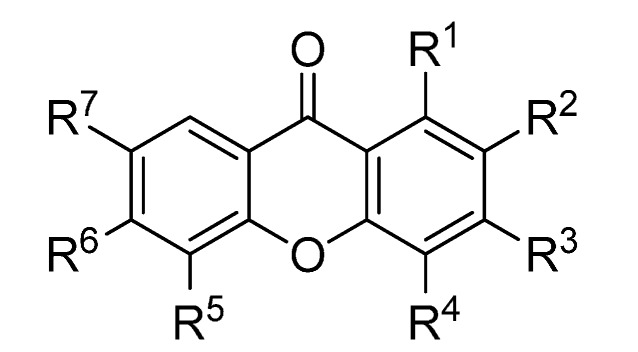
Chemical structures of tested dioxygenated xanthones.

**Figure 3:**
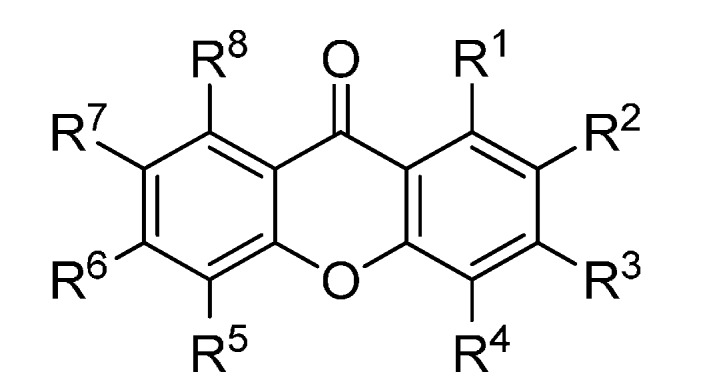
Chemical structures of tested trioxygenated xanthones.

**Figure 4:**
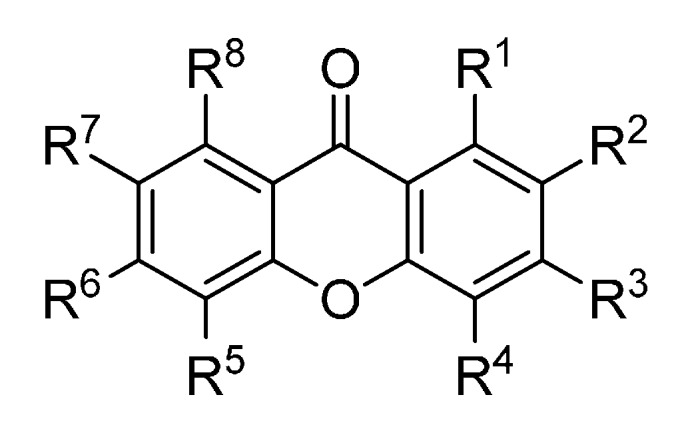
Chemical structures of tested tetraoxygenated xanthones.

**Figure 5:**
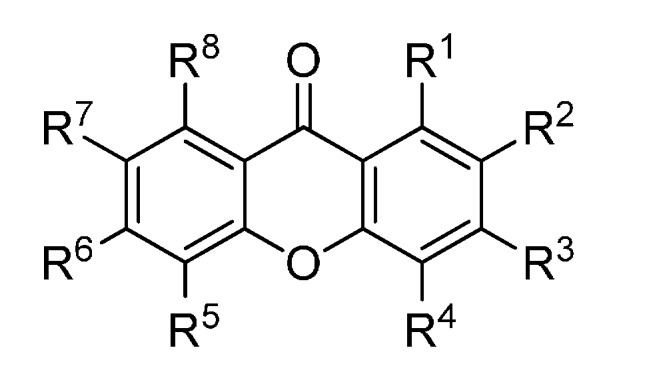
Chemical structures of tested pentaoxygenated xanthones.

**Figure 6:**
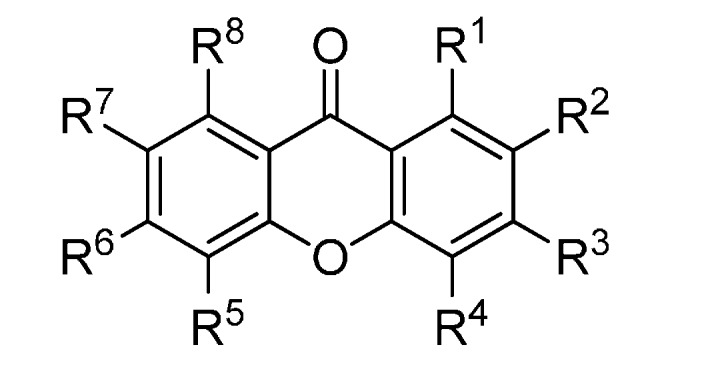
Chemical structures of tested hexaoxygenated xanthones.

**Figure 7:**
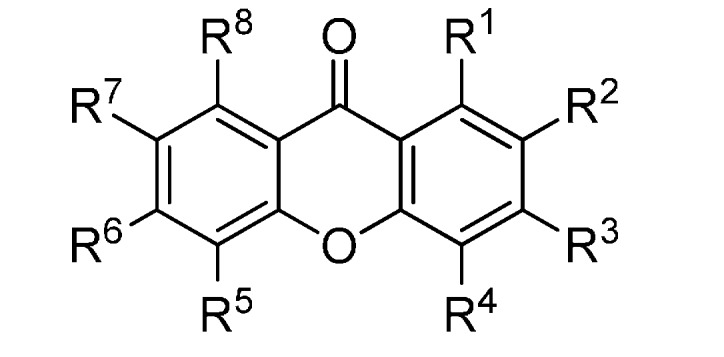
Chemical structures of tested tetraoxygenated and prenylated xanthones. A = 1,1-dimethylallyl; P = Prenyl; MOB = 3-hydroxy-3-methoxybutyl.

**Figure 8:**
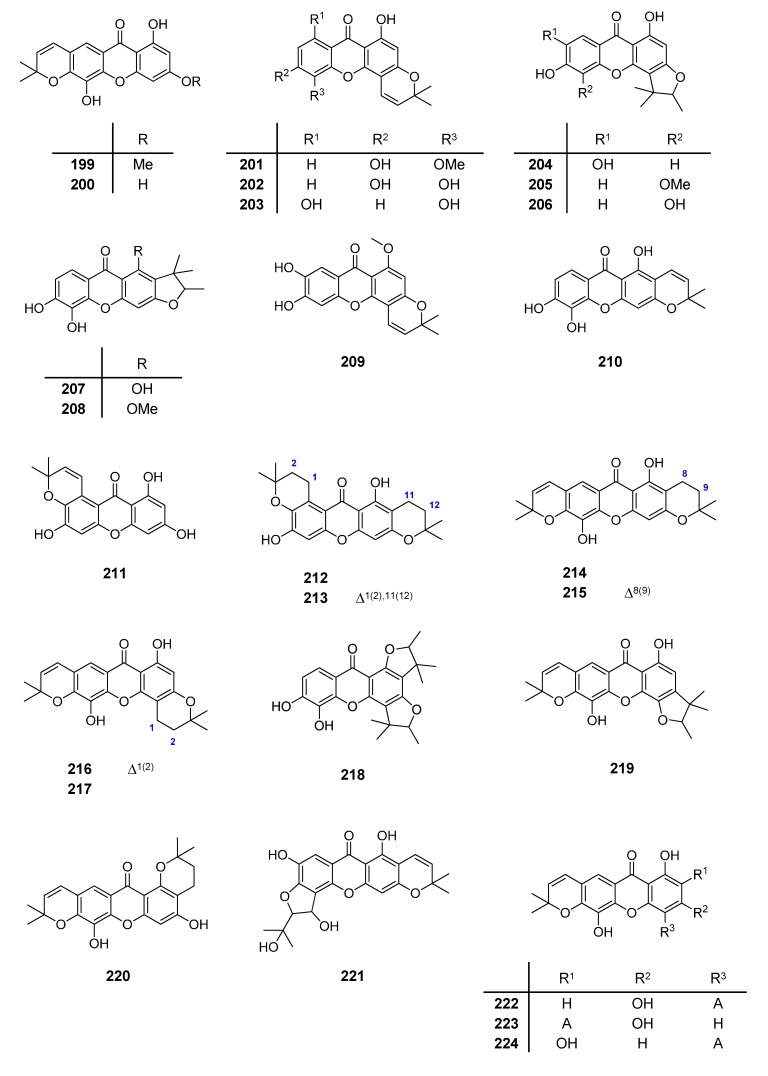

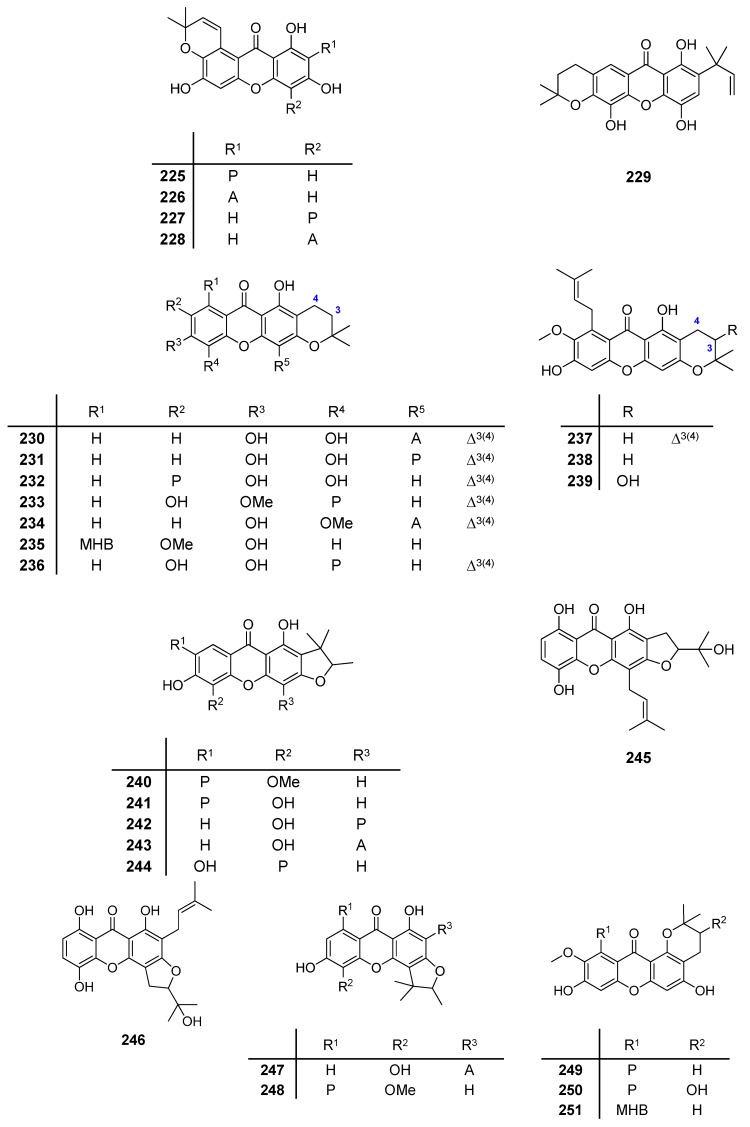

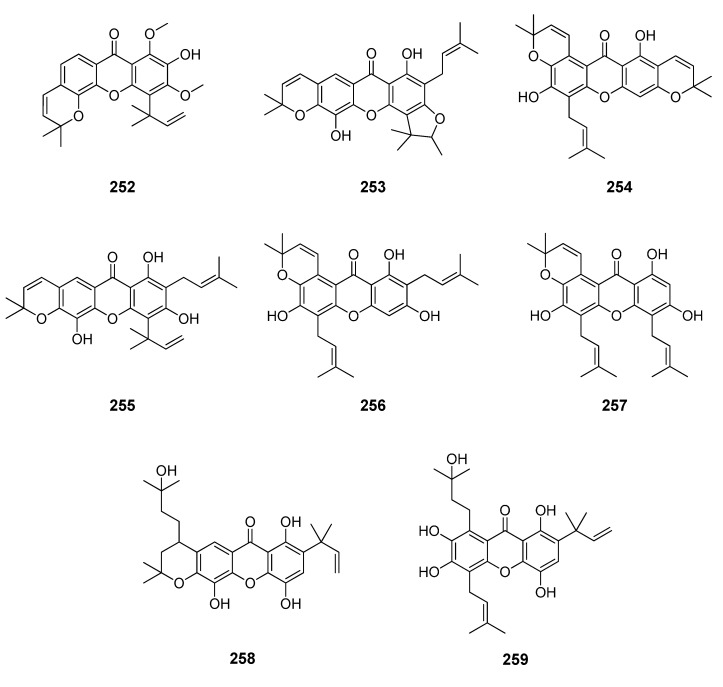
Chemical structures of tested tetraoxygenated and prenylated xanthones and cyclic derivatives.

**Figure 9:**
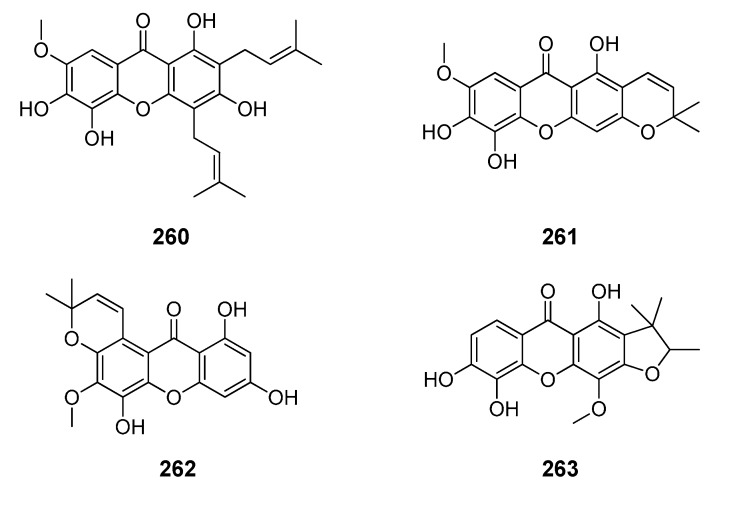
Chemical structures of tested pentaoxygenated and prenylated xanthones.

**Figure 10:**
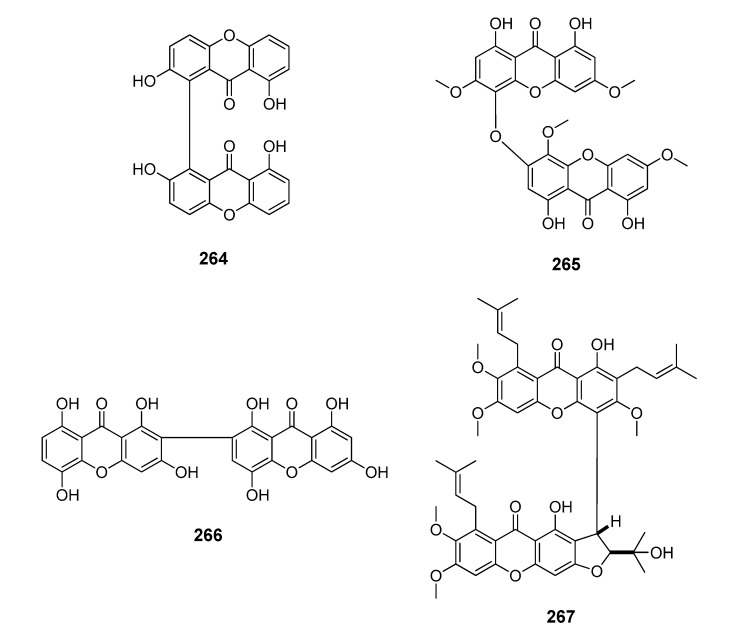
Chemical structures of tested dimeric xanthones.

**Figure 11:**
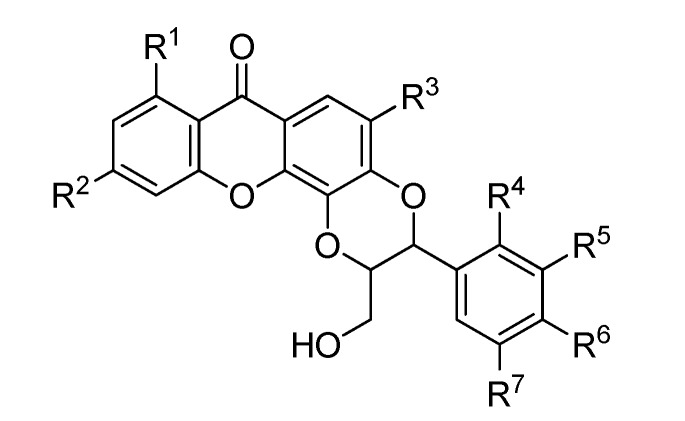
Chemical structures of tested derivatives from xanthones.

A set of natural xanthones with 272 structures was assembled from literature reports. This set consists of 19.9% antifungal xanthones [12,13,14,17]. The remaining structures were collected from existing reviews [21,22]. Their IUPAC names are displayed in the Supplementary Material. For comparative purposes, xanthones were divided into ten classes according to their substitution features: monooxygenated (MX), dioxygenated (DX), trioxygenated (TrX), tetraoxygenated (TeX), pentaoxygenated (PeX), hexaoxygenated (HX), tetraoxygented with prenyls and their cyclic derivatives (TePX), pentaoxygenated with prenyls (PePX), dimers (Dim) and xanthone derivatives (XD). All structures are shown in Figure 1, Figure 2, Figure 3, Figure 4, Figure 5, Figure 6, Figure 7, Figure 8, Figure 9, Figure 10 and Figure 11.

**Table:** 

	R^1^	R^2^	R^3^	R^4^	R^5^
**1**	H	H	H	H	H
**2**	OH	H	H	H	H
**3**	H	OH	H	H	H
**4**	H	H	OH	H	H
**5**	H	H	H	OH	H
**6**	H	H	H	H	OH
**7**	OMe	H	H	H	H
**8**	H	OMe	H	H	H
**9**	H	H	OMe	H	H
**10**	H	H	H	OMe	H

**Table:** 

	R^1^	R^2^	R^3^	R^4^	R^5^	R^6^	R^7^
**11**	OH	OH	H	H	H	H	H
**12**	H	OH	OH	H	H	H	H
**13**	H	H	OH	OH	H	H	H
**14**	H	H	OH	H	OH	H	H
**15**	H	H	OH	H	H	OH	H
**16**	OH	H	H	H	H	H	OH
**17**	OH	H	H	H	OH	H	H
**18**	H	H	OH	OMe	H	H	H
**19**	H	H	OMe	OH	H	H	H
**20**	H	H	OH	H	OMe	H	H
**21**	OMe	OH	H	H	H	H	H
**22**	H	OMe	OH	H	H	H	H
**23**	OH	H	H	H	OMe	H	H
**24**	OMe	H	H	H	OH	H	H
**25**	OH	H	H	H	H	H	OMe
**26**	OMe	OMe	H	H	H	H	H
**27**	H	OMe	OMe	H	H	H	H
**28**	H	H	OMe	OMe	H	H	H
**29**	H	H	OMe	H	OMe	H	H
**30**	OMe	H	OMe	H	H	H	H
**31**	H	OMe	H	H	H	H	OMe

**Table:** 

	R^1^	R^2^	R^3^	R^4^	R^5^	R^6^	R^7^	R^8^
**32**	OMe	OH	H	H	H	H	H	OMe
**33**	OMe	OMe	OH	H	H	H	OMe	H
**34**	OH	H	OMe	H	OH	H	H	H
**35**	OH	H	OH	H	OMe	OH	H	H
**36**	OH	H	OH	H	OMe	OMe	H	H
**37**	OH	H	H	H	OH	OMe	H	H

**Table:** 

	R^1^	R^2^	R^3^	R^4^	R^5^	R^6^	R^7^	R^8^
**38**	OH	OMe	OMe	H	OMe	H	H	H
**39**	OH	OMe	OMe	H	H	H	OMe	H
**40**	OH	H	OMe	OMe	OMe	H	H	H
**41**	OH	H	OMe	OMe	H	H	OMe	H
**42**	OH	H	OMe	H	OMe	OMe	H	H
**43**	OH	H	OMe	H	OMe	H	H	OMe
**44**	OH	H	OMe	H	H	OMe	OMe	H
**45**	OH	H	OMe	H	H	OMe	H	OMe
**46**	OH	H	OMe	H	H	H	OMe	OMe
**47**	OMe	OH	OMe	H	H	H	OMe	H
**48**	H	OH	H	H	OMe	OMe	OMe	H
**49**	OMe	OMe	OH	OMe	H	H	H	H
**50**	OMe	OMe	OH	H	H	H	OMe	H
**51**	OMe	H	OH	H	OMe	OMe	H	H
**52**	OMe	H	OH	H	H	H	OMe	OMe
**53**	H	OMe	OMe	OH	H	OMe	H	H
**54**	OMe	OMe	OMe	H	OH	H	H	H
**55**	H	H	OH	OMe	H	OMe	H	OMe
**56**	OMe	OMe	OMe	H	H	H	OH	H
**57**	OMe	H	OMe	H	H	H	OH	OMe
**58**	OMe	H	OMe	H	OMe	H	H	OH
**59**	OH	OMe	OH	H	OH	H	H	H
**60**	OH	OH	H	H	OMe	OMe	H	H
**61**	OH	OMe	OH	H	OMe	H	H	H
**62**	OH	OMe	OH	H	H	H	OMe	H
**63**	OH	OMe	OH	H	H	H	H	OMe
**64**	OH	H	OH	OMe	OMe	H	H	H
**65**	OH	H	OH	OMe	H	H	OMe	H
**66**	OH	H	OH	H	OMe	OMe	H	H
**67**	OH	H	OH	H	OMe	H	H	OMe
**68**	OH	H	OH	H	H	H	H	H
**69**	OH	H	OH	H	H	H	H	H
**70**	OH	H	OMe	OH	OMe	H	H	H
**71**	OH	OMe	OMe	H	OH	H	H	H
**72**	OH	H	OMe	OMe	OH	H	H	H
**73**	OH	H	OMe	H	OH	H	OMe	H
**74**	OH	H	OMe	H	OH	H	H	OMe
**75**	OH	H	H	H	OH	OMe	OMe	H
**76**	OH	H	OMe	H	OMe	OH	H	H
**77**	OH	H	OMe	H	H	OH	OMe	H
**78**	OH	H	H	H	OMe	OH	OMe	H
**79**	OH	H	H	H	H	OH	OMe	OMe
**80**	OH	H	H	H	H	OH	-CH_2_-	
**81**	OH	OMe	OMe	H	H	H	OH	H
**82**	OH	H	OMe	OMe	H	H	OH	H
**83**	OH	H	OMe	H	OMe	H	OH	H
**84**	OH	H	OMe	H	H	OMe	OH	H
**85**	OH	H	OMe	H	H	H	OH	OMe
**86**	OH	OMe	H	H	H	H	OMe	OH
**87**	OH	H	OMe	H	OMe	H	H	OH
**88**	OH	H	OMe	H	H	OMe	H	OH
**89**	OH	H	OMe	H	H	OMe	H	OH
**90**	H	OH	OMe	OH	H	OMe	H	H
**91**	OMe	OH	H	H	OH	OMe	H	H
**92**	OMe	OH	H	H	OMe	OH	H	H
**93**	OMe	OH	H	H	H	H	OH	OMe
**94**	OMe	OMe	OH	H	H	H	H	OH
**95**	OMe	H	OH	H	H	H	OH	OMe
**96**	OMe	H	OH	H	OMe	OH	H	H
**97**	OMe	H	OH	H	H	H	OMe	OH
**98**	OMe	H	OMe	OH	H	OH	H	H
**99**	OMe	H	OMe	H	OH	OH	H	H
**100**	OH	OH	OH	H	OMe	H	H	H
**101**	OH	OH	OMe	H	H	H	H	OH
**102**	OH	OMe	OH	H	OH	H	H	H
**103**	OH	H	OH	H	OH	OMe	H	H
**104**	OH	H	OH	H	OMe	OH	H	H
**105**	OH	H	OH	H	H	OH	OMe	H
**106**	OH	H	OH	H	H	OMe	OH	H
**107**	OH	H	OH	H	H	H	OH	OMe
**108**	OH	H	OH	H	OMe	H	H	OH
**109**	OH	H	OH	H	H	H	OMe	OH
**110**	OH	H	OMe	OH	H	H	OH	H
**111**	OH	H	H	OH	H	H	OH	OMe
**112**	OH	H	OMe	OH	H	H	H	OH
**113**	OH	H	H	OH	H	OMe	H	OH
**114**	OH	H	OMe	H	OH	OH	H	H
**115**	OH	H	OMe	H	OH	H	OH	H
**116**	OH	H	OMe	H	H	H	OH	H
**117**	OH	OMe	H	H	H	OH	H	OH
**118**	OH	H	OMe	H	H	H	OH	OH
**119**	OH	H	H	H	H	OMe	OH	OH
**120**	OMe	OH	H	OH	OH	H	H	H
**121**	OMe	H	OH	H	OH	OH	H	H
**122**	OH	H	OH	H	OH	OH	H	H
**123**	OH	H	OH	H	OH	H	OH	H
**124**	OH	H	OH	H	OH	H	H	OH
**125**	OH	H	OH	H	H	OH	OH	H
**126**	OH	H	OH	H	H	H	OH	OH
**127**	OMe	-CH_2_-		H	H	H	H	H
**128**	OMe	-CH_2_-		H	H	H	H	H
**129**	OMe	OMe	OMe	H	H	H	OMe	H
**130**	OMe	OMe	H	H	H	OMe	H	OMe
**131**	OMe	H	OMe	OMe	OMe	H	H	H
**132**	OMe	H	OMe	OMe	H	H	OMe	H
**133**	OMe	H	OMe	H	OMe	H	H	OMe
**134**	OMe	H	OMe	H	H	OMe	OMe	H
**135**	H	OMe	OMe	OMe	OMe	H	H	H

**Table:** 

	R^1^	R^2^	R^3^	R^4^	R^5^	R^6^	R^7^	R^8^
**136**	OH	OMe	OMe	OMe	OMe	H	H	H
**137**	OH	OMe	OMe	OMe	H	H	OMe	H
**138**	OH	H	OMe	OMe	H	H	OMe	OMe
**139**	OH	H	OMe	H	OMe	OMe	OMe	H
**140**	OH	H	OMe	H	OMe	H	OMe	OMe
**141**	OH	H	OMe	OMe	H	H	OH	OMe
**142**	OH	H	OH	OMe	OMe	H	H	OH
**143**	OH	H	OH	H	H	OH	OMe	OMe

**Table:** 

	R^1^	R^2^	R^3^	R^4^	R^5^	R^6^	R^7^	R^8^
**144**	OH	H	OMe	H	OMe	OMe	OMe	OMe
**145**	OH	OMe	OMe	OMe	H	OMe	H	OH
**146**	OH	H	OMe	H	OMe	OMe	OMe	OH
**147**	OMe	OMe	OMe	OMe	H	OMe	OMe	H

**Table:** 

	R^1^	R^2^	R^3^	R^4^	R^5^	R^6^	R^7^	R^8^
**148**	OH	P	OH	H	OH	OH	H	H
**149**	OH	H	OH	P	OH	OH	H	H
**150**	OH	P	OH	H	OMe	OMe	H	H
**151**	OH	P	OH	H	OMe	OH	H	H
**152**	OH	H	OH	H	H	OH	OH	P
**153**	OH	P	OMe	H	OH	H	H	OH
**154**	OH	P	OH	H	OH	H	H	OH
**155**	OH	H	OH	P	OH	H	H	OH
**156**	OH	H	H	OH	OMe	OH	P	H
**157**	P	OMe	OH	OH	H	H	H	OH
**158**	OH	A	H	OH	OH	OH	H	H
**159**	OH	A	OMe	H	OH	OH	H	H
**160**	OH	H	OH	A	H	OH	OH	H
**161**	OH	OH	H	A	OH	OH	H	H
**162**	OMe	OH	H	A	OH	OH	H	H
**163**	OH	MOB	OH	H	OH	OH	H	H
**164**	OH	P	OH	H	P	OH	OMe	H
**165**	OH	P	OH	H	OMe	OH	P	H
**166**	OH	P	OH	H	H	OH	OH	P
**167**	OH	P	OMe	H	H	OH	OMe	P
**168**	OH	P	OH	H	H	OH	OMe	P
**169**	OH	P	OH	P	OH	OH	H	H
**170**	OH	P	OH	H	OH	OH	P	H
**171**	OH	P	OH	P	OH	H	H	OH
**172**	OH	P	OH	P	OH	H	H	OH
**173**	OH	P	OMe	H	H	OH	OMe	P
**174**	OH	P	OH	H	H	OH	OMe	P
**175**	OH	P	OMe	H	H	OMe	OMe	P
**176**	OH	P	H	H	H	H	OMe	P
**177**	OH	P	H	H	H	H	OMe	P
**178**	OH	P	H	H	H	H	OMe	P
**179**	OH	P	H	H	H	H	OMe	P
**180**	OH	P	H	H	H	H	OMe	P
**181**	OH	P	H	H	H	H	OMe	P
**182**	OH	P	H	H	H	H	OMe	P
**183**	OH	P	OH	H	H	OH	OMe	MOB
**184**	OH	MOB	OH	H	H	OH	OMe	P
**185**	OH	P	OH	H	H	OH	OH	MOB
**186**	OH	P	OH	A	OH	OH	H	H
**187**	OH	OH	H	A	OH	OH	P	H
**188**	OH	H	OH	A	OH	OH	P	H
**189**	OH	A	OH	H	P	OH	OH	H
**190**	OH	A	OH	H	OH	OH	H	P
**191**	OH	H	OH	A	H	OH	OMe	P
**192**	OH	A	OH	H	OMe	OH	P	H
**193**	OH	P	OH	H	H	OH	OMe	MOB
**194**	OH	P	OH	H	P	OH	OH	P
**195**	OH	P	OH	H	P	OH	OMe	P
**196**	OH	A	H	OH	OH	OH	P	P
**197**	OH	A	H	OH	P	OH	OH	P
**198**	OH	A	OH	P	OH	OH	H	P

**Table:** 

	R^1^	R^2^	R^3^	R^4^	R^5^	R^6^	R^7^
**268**	OH	H	OMe	H	H	OMe	OH
**269**	OH	H	OMe	OMe	H	OMe	OH
**270**	OH	H	OMe	H	OMe	OH	OMe
**271**	OMe	H	OMe	H	OMe	OH	OMe
**272**	OH	OH	H	H	OMe	OH	OMe

Each ligand was independently submitted to AM1 conformational search, and the minimum energy conformer was then DFT optimized at B3LYP/6-31G* level. Each optimized structure was docked into the active site of each receptor using AutoDock Vina. General structural information for the proteins employed in the present study is shown in Table 1.

**Table 1:** Tested receptor data.

ID	Enzyme	PDB Code	Resolution (Å)	Source	Ref.
R1	gp120	4DKR	1.80	HIV-1	[42]
R2	Reverse transcriptase	2WON	2.80	HIV-1	[43]
R3	Ribonuclease F1	1FUT	2.00	*Fusarium moniliforme*	[44]
R4	Cytochrome P450 14α-sterol demethylase	1EA1	2.21	*Mycobacterium tuberculosis*	[45]
R5	α-l-Arabinofuranosidase	1QW9	1.20	*Geobacillus stearothermophilus*	[46]
R6	α-Fucosidase	1ODU	2.80	*Thermotoga maritime*	[35]
R7	Nitric oxide reductase	3AYG	2.70	*Geobacillus stearothermophilus*	[47]
R8	*N*-myristoyltransferase	1IYK	2.30	*Candida albicans*	[48]
R9	*N*-myristoyltransferase	1IYL	3.20	*Candida albicans*	[48]
R10	Trichodiene synthase	1YYR	2.50	*Fusarium sporotrichioides*	[49]

Affinity energies for each ligand—receptor complex were compiled as a data matrix summarized in Table 2. Lowest predicted energy for each protein target is highlighted in bold text. From these, the TePX resulted to be responsible for the strongest ligand—receptor complexes to eight enzymes (**215**/**223**, **214**, **254**, **215**, **225**, **220**, **256** and **256** for R2–R9 respectively). R1 was markedly complexed by Dim **266** while R10 was strongly non-covalent bonded by TeX **116**. This fact let to infer significant differences in structural interactions among enzymes R1 and R10 regarding to the rest. None of xanthones were able to establish a stronger complex than that of co-crystalized ligand (CCI) with R1. For R2–R10, a variable number of xanthones exhibited affinity energies lower than those for CCIs (ranging on 11.8%–87.9% depending on the respective target).

**Table 2:** Affinity energies ^a^ from docking of xanthones with target enzymes.

ID	Type	Affinity Energy (kcal/mol)									
		R1	R2	R3	R4	R5	R6	R7	R8	R9	R10
**1**	MX	−7.8	−10.7	−5.9	−8.6	−6.7	−7.2	−7.8	−7.9	−8.8	−8.3
**2**	MX	−6.9	−10.7	−6.3	−8.9	−7.4	−8.0	−8.3	−8.4	−9.0	−8.6
**3**	MX	−7.8	−11.2	−6.2	−8.8	−7.3	−8.1	−7.9	−8.1	−9.1	−8.6
**4**	MX	−7.2	−10.6	−6.2	−9.0	−7.1	−7.5	−8.0	−7.6	−8.7	−8.5
**5**	MX	−7.2	−10.9	−6.0	−8.7	−7.3	−7.4	−8.4	−8.0	−8.9	−8.7
**6**	MX	−7.0	−10.9	−6.0	−8.7	−7.3	−7.4	−8.4	−7.9	−8.9	−8.6
**7**	MX	−5.4	−10.7	−5.9	−9.1	−7.0	−7.2	−8.0	−8.2	−8.1	−8.2
**8**	MX	−7.1	−11.4	−5.9	−9.1	−7.2	−7.9	−7.8	−7.9	−9.2	−7.8
**9**	MX	−7.4	−10.6	−5.8	−9.1	−7.1	−7.3	−7.6	−8.0	−8.9	−7.8
**10**	MX	−5.8	−10.7	−6.0	−9.2	−7.1	−7.8	−8.4	−7.4	−8.8	−8.8
**11**	DX	−7.0	−11.3	−6.1	−9.0	−7.8	−8.2	−8.1	−8.4	−9.3	−8.6
**12**	DX	−7.4	−11.1	−6.3	−9.1	−7.6	−8.3	−8.2	−8.0	−9.0	−9.0
**13**	DX	−6.6	−10.8	−6.4	−8.9	−7.7	−7.7	−8.3	−7.7	−8.9	−8.5
**14**	DX	−7.3	−10.6	−6.7	−9.0	−7.3	−7.8	−8.0	−7.7	−8.9	−8.7
**15**	DX	−6.7	−10.3	−6.1	−8.9	−7.1	−7.3	−7.8	−7.6	−8.7	−8.4
**16**	DX	−6.8	−10.4	−6.4	−9.0	−7.3	−7.9	−8.3	−7.8	−8.8	−9.0
**17**	DX	−6.1	−10.7	−6.4	−9.5	−7.2	−8.3	−8.0	−8.3	−8.8	−8.6
**18**	DX	−5.4	−10.4	−6.3	−9.1	−7.5	−7.2	−8.4	−7.3	−8.8	−8.4
**19**	DX	−6.4	−10.6	−5.9	−9.1	−7.1	−7.8	−7.3	−7.9	−9.1	−8.3
**20**	DX	−6.1	−10.7	−6.3	−9.0	−7.2	−7.0	−8.1	−7.8	−8.9	−8.5
**21**	DX	−5.3	−10.4	−5.9	−9.1	−7.6	−7.1	−7.8	−7.6	−8.0	−8.4
**22**	DX	−6.4	−11.2	−5.7	−9.4	−7.2	−8.2	−8.1	−7.7	−8.8	−8.4
**23**	DX	−5.4	−10.6	−6.4	−9.3	−7.3	−8.1	−8.1	−7.9	−8.8	−8.4
**24**	DX	−5.7	−10.9	−6.0	−9.4	−7.4	−8.2	−8.1	−8.4	−8.5	−8.6
**25**	DX	−5.8	−10.8	−6.1	−8.9	−7.3	−7.9	−8.3	−7.9	−9.0	−8.0
**26**	DX	−4.9	−10.8	−5.9	−9.3	−7.0	−7.1	−7.6	−7.4	−8.2	−7.3
**27**	DX	−7.2	−10.5	−5.9	−8.9	−6.8	−7.0	−7.4	−7.9	−8.8	−7.5
**28**	DX	−5.5	−10.3	−5.9	−9.2	−7.0	−6.9	−7.3	−7.5	−9.0	−7.8
**29**	DX	−5.9	−10.8	−6.0	−8.9	−7.1	−7.3	−8.1	−7.4	−9.0	−7.4
**30**	DX	−5.2	−10.6	−5.9	−9.0	−6.9	−7.1	−7.3	−8.0	−8.5	−8.1
**31**	DX	−7.2	−10.9	−5.6	−9.1	−7.1	−7.8	−7.1	−7.8	−9.2	−7.3
**32**	TrX	−5.3	−10.7	−6.0	−8.9	−7.4	−7.6	−7.8	−7.4	−8.1	−8.4
**33**	TrX	−5.1	−10.4	−5.6	−9.4	−7.0	−7.7	−7.2	−7.8	−8.4	−7.4
**34**	TrX	−6.0	−10.4	−6.3	−8.8	−7.3	−7.6	−7.8	−7.8	−8.8	−8.7
**35**	TrX	−5.8	−10.5	−6.5	−8.9	−7.1	−8.1	−8.3	−7.6	−8.8	−8.4
**36**	TrX	−5.5	−10.1	−6.0	−9.3	−7.1	−8.0	−7.8	−7.8	−8.8	−8.0
**37**	TrX	−6.4	−10.6	−6.5	−9.2	−7.7	−8.0	−7.8	−8.1	−8.9	−8.3
**38**	TeX	−5.3	−9.9	−6.3	−8.8	−7.1	−7.5	−7.8	−7.4	−9.0	−7.3
**39**	TeX	−5.2	−10.3	−6.0	−9.1	−7.3	−7.4	−6.8	−8.0	−9.1	−6.9
**40**	TeX	−5.4	−9.9	−6.4	−8.5	−6.8	−6.9	−6.9	−7.6	−8.9	−8.4
**41**	TeX	−5.3	−9.7	−5.9	−9.1	−6.9	−7.6	−7.6	−7.8	−9.1	−7.2
**42**	TeX	−5.2	−10.0	−5.9	−9.1	−7.1	−7.5	−7.3	−7.7	−8.9	−7.1
**43**	TeX	−5.9	−10.2	−6.3	−8.7	−7.1	−7.7	−7.3	−7.7	−8.8	−8.8
**44**	TeX	−5.5	−9.9	−5.9	−8.5	−7.3	−7.6	−7.5	−7.7	−8.7	−7.4
**45**	TeX	−5.7	−9.5	−6.1	−8.6	−7.3	−7.6	−7.4	−7.6	−8.6	−6.7
**46**	TeX	−5.3	−9.5	−5.7	−9.3	−6.8	−7.6	−7.9	−7.6	−8.7	−7.6
**47**	TeX	−5.2	−10.3	−5.8	−9.2	−7.0	−7.1	−7.0	−7.9	−8.5	−8.1
**48**	TeX	−5.0	−9.3	−6.0	−8.9	−6.8	−7.4	−7.1	−7.7	−8.6	−6.6
**49**	TeX	−5.1	−11.0	−5.9	−8.8	−6.7	−6.7	−7.0	−7.5	−8.2	−7.4
**50**	TeX	−5.3	−11.2	−5.8	−9.4	−6.9	−7.3	−8.1	−7.8	−8.6	−7.5
**51**	TeX	−5.4	−9.6	−5.9	−9.2	−6.8	−7.6	−7.8	−7.9	−8.5	−6.5
**52**	TeX	−5.4	−10.7	−6.4	−9.4	−6.9	−7.9	−8.1	−7.8	−8.4	−7.0
**53**	TeX	−5.8	−10.1	−5.9	−8.7	−7.1	−7.2	−6.7	−7.6	−8.9	−7.3
**54**	TeX	−5.2	−10.2	−5.9	−9.1	−7.1	−6.9	−7.7	−7.6	−8.3	−6.7
**55**	TeX	−5.4	−9.5	−6.1	−9.1	−7.2	−7.5	−7.8	−7.6	−8.2	−7.2
**56**	TeX	−5.1	−10.1	−6.2	−9.2	−6.8	−7.2	−7.4	−7.8	−8.3	−6.6
**57**	TeX	−5.3	−9.8	−5.9	−9.1	−7.2	−6.4	−7.1	−7.8	−8.3	−8.0
**58**	TeX	−5.6	−9.9	−6.2	−8.7	−6.9	−7.1	−8.5	−7.4	−8.7	−7.8
**59**	TeX	−5.6	−10.5	−6.1	−9.4	−7.0	−9.1	−7.9	−7.8	−8.8	−8.9
**60**	TeX	−5.3	−10.3	−5.9	−9.4	−7.6	−8.4	−7.7	−8.0	−9.1	−8.1
**61**	TeX	−5.3	−10.5	−6.3	−9.3	−7.9	−8.2	−7.9	−7.9	−8.9	−8.4
**62**	TeX	−5.4	−10.4	−5.9	−9.3	−7.2	−8.7	−8.2	−7.8	−9.0	−7.3
**63**	TeX	−5.4	−9.8	−5.8	−8.9	−7.4	−8.8	−8.3	−7.7	−8.7	−8.8
**64**	TeX	−5.8	−10.6	−6.3	−8.2	−8.2	−6.9	−7.3	−7.3	−8.8	−7.9
**65**	TeX	−5.6	−10.4	−6.4	−9.1	−7.3	−7.5	−8.1	−7.6	−9.0	−7.8
**66**	TeX	−5.6	−9.9	−6.2	−9.3	−7.6	−7.7	−7.8	−8.1	−8.7	−7.8
**67**	TeX	−6.0	−10.3	−6.2	−8.9	−7.4	−7.6	−7.6	−8.1	−8.4	−8.1
**68**	TeX	−5.6	−9.5	−6.2	−8.9	−7.4	−7.4	−7.5	−8.0	−8.7	−7.3
**69**	TeX	−5.3	−10.4	−6.1	−9.4	−7.2	−8.1	−8.1	−7.9	−8.5	−7.4
**70**	TeX	−5.5	−10.1	−6.3	−8.5	−7.2	−7.9	−7.1	−7.6	−9.1	−8.3
**71**	TeX	−5.4	−9.7	−6.1	−9.2	−7.1	−7.1	−7.8	−7.7	−9.0	−7.5
**72**	TeX	−5.4	−9.7	−6.2	−8.9	−6.9	−7.2	−7.0	−7.6	−9.0	−7.6
**73**	TeX	−5.7	−9.9	−6.1	−8.9	−7.3	−7.6	−7.4	−7.8	−8.9	−8.4
**74**	TeX	−5.7	−9.7	−6.3	−8.5	−7.2	−7.6	−8.0	−7.6	−8.4	−8.9
**75**	TeX	−5.6	−9.8	−6.6	−9.1	−7.6	−7.4	−7.5	−7.9	−8.5	−8.1
**76**	TeX	−5.5	−10.0	−6.5	−9.0	−7.1	−7.6	−7.8	−7.5	−8.9	−8.7
**77**	TeX	−5.8	−10.1	−6.3	−9.0	−7.4	−8.1	−7.6	−7.9	−8.8	−9.0
**78**	TeX	−5.9	−9.9	−6.0	−8.9	−7.3	−7.6	−7.4	−7.7	−8.4	−8.5
**79**	TeX	−5.5	−9.9	−6.3	−9.2	−7.2	−7.3	−7.9	−7.7	−8.5	−7.2
**80**	TeX	−6.1	−10.5	−6.7	−9.6	−8.3	−8.8	−8.9	−8.7	−8.9	−9.1
**81**	TeX	−5.4	−10.2	−6.4	−9.2	−7.1	−8.0	−7.4	−7.8	−9.0	−6.8
**82**	TeX	−5.3	−9.5	−6.3	−9.0	−6.9	−7.6	−7.6	−7.8	−9.0	−7.5
**83**	TeX	−5.6	−10.1	−6.5	−8.7	−7.1	−7.5	−7.9	−7.9	−8.8	−8.4
**84**	TeX	−5.6	−10.2	−6.1	−8.8	−7.2	−7.7	−7.5	−7.7	−9.1	−7.5
**85**	TeX	−5.5	−9.7	−6.2	−8.9	−7.6	−7.6	−7.7	−7.7	−8.5	−9.0
**86**	TeX	−5.5	−9.6	−6.1	−9.1	−7.1	−8.4	−7.3	−8.2	−8.6	−7.7
**87**	TeX	−5.6	−9.7	−6.6	−8.6	−7.2	−7.6	−8.4	−7.7	−8.7	−8.9
**88**	TeX	−5.5	−9.6	−6.1	−8.7	−7.2	−7.6	−7.8	−7.5	−8.6	−7.2
**89**	TeX	−5.7	−9.7	−6.1	−9.2	−7.0	−8.0	−7.9	−8.1	−8.7	−8.1
**90**	TeX	−6.5	−10.2	−6.2	−9.5	−7.1	−8.0	−7.6	−7.8	−9.2	−7.3
**91**	TeX	−5.7	−9.6	−6.1	−8.9	−7.6	−7.7	−8.0	−7.9	−8.5	−7.7
**92**	TeX	−5.6	−10.0	−6.1	−9.3	−7.0	−7.3	−8.2	−7.5	−7.9	−7.8
**93**	TeX	−5.6	−9.9	−6.0	−8.6	−7.3	−7.3	−7.8	−7.4	−8.1	−7.9
**94**	TeX	−5.3	−9.8	−6.1	−9.3	−7.1	−7.1	−8.1	−7.7	−8.8	−7.2
**95**	TeX	−5.5	−9.9	−6.4	−8.9	−7.5	−7.4	−8.2	−8.0	−8.3	−8.0
**96**	TeX	−5.5	−10.2	−6.1	−9.0	−7.1	−7.4	−7.8	−7.8	−8.3	−7.9
**97**	TeX	−5.7	−10.2	−6.0	−9.1	−7.3	−8.0	−8.2	−8.1	−8.4	−7.7
**98**	TeX	−5.3	−9.8	−6.5	−8.7	−7.7	−7.5	−6.9	−7.8	−8.6	−7.7
**99**	TeX	−5.6	−9.8	−6.2	−8.8	−7.9	−7.5	−7.7	−8.0	−8.6	−7.5
**100**	TeX	−5.6	−10.7	−6.4	−9.4	−8.0	−8.8	−7.9	−8.2	−9.1	−9.1
**101**	TeX	−5.6	−9.9	−6.4	−9.6	−7.1	−8.1	−7.8	−7.8	−8.6	−8.4
**102**	TeX	−5.6	−10.3	−6.2	−9.4	−7.3	−9.1	−7.8	−7.7	−8.9	−8.8
**103**	TeX	−5.6	−9.7	−6.6	−9.1	−8.1	−8.2	−7.8	−8.1	−8.8	−7.9
**104**	TeX	−5.6	−10.2	−6.5	−8.8	−7.6	−7.6	−7.9	−7.9	−8.8	−8.3
**105**	TeX	−5.9	−9.7	−6.3	−9.1	−7.6	−8.0	−8.0	−7.9	−8.8	−8.8
**106**	TeX	−5.7	−10.6	−6.2	−9.0	−7.7	−7.7	−7.4	−8.0	−8.9	−7.8
**107**	TeX	−5.5	−10.3	−6.1	−8.9	−7.2	−8.2	−8.1	−7.9	−8.5	−8.6
**108**	TeX	−5.6	−9.6	−6.6	−9.1	−7.6	−7.5	−8.4	−8.1	−8.4	−8.4
**109**	TeX	−6.0	−10.1	−6.6	−9.0	−8.0	−8.2	−8.1	−7.9	−8.6	−7.9
**110**	TeX	−5.8	−9.7	−6.3	−8.9	−7.5	−7.7	−7.5	−7.8	−9.1	−8.7
**111**	TeX	−5.7	−10.0	−6.5	−9.2	−7.5	−7.7	−8.6	−7.7	−8.3	−8.5
**112**	TeX	−5.9	−9.8	−6.5	−8.7	−7.6	−8.0	−7.4	−7.9	−8.8	−8.4
**113**	TeX	−5.7	−10.0	−6.6	−8.7	−7.6	−7.6	−8.3	−7.8	−8.4	−8.9
**114**	TeX	−5.5	−9.8	−6.5	−8.8	−7.9	−7.6	−7.6	−8.0	−8.9	−9.1
**115**	TeX	−6.2	−10.4	−6.4	−8.8	−7.9	−7.7	−7.9	−8.0	−8.7	−8.7
**116**	TeX	−5.8	−9.7	−6.5	−9.1	−7.6	−8.0	−7.5	−8.2	−8.9	**−9.3**
**117**	TeX	−5.5	−10.1	−6.9	−9.0	−8.0	−8.2	−8.1	−7.9	−8.6	−7.9
**118**	TeX	−6.3	−10.1	−6.5	−9.1	−7.6	−8.7	−7.8	−7.9	−8.5	−9.1
**119**	TeX	−5.8	−9.9	−6.4	−9.6	−7.1	−8.1	−7.8	−7.7	−8.5	−8.4
**120**	TeX	−5.6	−10.1	−6.3	−8.7	−7.7	−8.2	−7.4	−7.7	−8.2	−8.4
**121**	TeX	−5.7	−9.9	−6.3	−8.9	−7.8	−7.5	−8.2	−8.0	−8.4	−8.3
**122**	TeX	−5.6	−9.7	−6.7	−8.9	−8.0	−8.1	−8.3	−7.9	−8.7	−8.9
**123**	TeX	−6.1	−10.3	−6.4	−8.8	−7.8	−8.5	−7.9	−8.0	−8.5	−8.5
**124**	TeX	−5.7	−9.9	−6.7	−9.0	−7.8	−8.6	−8.4	−8.0	−8.4	−9.0
**125**	TeX	−5.7	−9.8	−6.4	−9.1	−7.7	−7.9	−8.0	−8.1	−8.8	−9.0
**126**	TeX	−6.3	−10.0	−6.7	−9.1	−7.9	−8.2	−8.1	−7.9	−8.5	−8.9
**127**	TeX	−5.5	−11.0	−6.5	−9.9	−7.5	−8.0	−7.5	−8.3	−9.3	−7.9
**128**	TeX	−5.5	−10.6	−6.2	−9.5	−7.5	−7.2	−7.9	−7.9	−8.5	−7.5
**129**	TeX	−4.9	−10.4	−5.5	−9.1	−6.9	−7.0	−6.4	−7.9	−8.3	−6.9
**130**	TeX	−5.1	−10.0	−5.7	−8.9	−7.1	−6.1	−7.0	−7.8	−8.4	−6.9
**131**	TeX	−5.4	−9.7	−6.0	−8.6	−6.7	−7.7	−6.6	−7.7	−8.7	−6.6
**132**	TeX	−5.3	−9.9	−5.9	−8.9	−6.9	−7.6	−6.7	−7.9	−9.0	−6.7
**133**	TeX	−5.9	−9.7	−6.1	−8.9	−7.1	−6.4	−7.2	−7.6	−8.1	−7.6
**134**	TeX	−5.1	−11.0	−5.9	−8.6	−7.3	−6.8	−6.6	−7.8	−8.5	−5.9
**135**	TeX	−5.2	−9.4	−6.1	−8.3	−7.1	−7.0	−6.5	−7.2	−8.6	−7.3
**136**	PeX	−5.4	−9.6	−6.4	−8.3	−6.6	−6.9	−6.7	−7.4	−9.0	−8.1
**137**	PeX	−5.1	−8.7	−5.9	−8.5	−7.1	−7.4	−6.8	−7.7	−9.1	−6.3
**138**	PeX	−5.2	−9.7	−5.8	−8.5	−6.8	−6.0	−7.0	−7.8	−8.9	−6.0
**139**	PeX	−5.1	−10.0	−6.0	−8.6	−7.3	−7.5	−7.2	−7.8	−8.6	−7.3
**140**	PeX	−5.3	−10.1	−6.0	−8.5	−7.1	−7.4	−7.4	−7.9	−8.2	−6.8
**141**	PeX	−5.5	−9.3	−5.9	−8.7	−7.3	−6.4	−7.0	−7.9	−8.2	−7.4
**142**	PeX	−6.0	−9.4	−6.6	−8.5	−8.0	−7.0	−7.4	−7.2	−8.3	−9.0
**143**	PeX	−5.7	−9.6	−6.0	−9.2	−7.4	−7.8	−8.0	−7.9	−8.5	−7.0
**144**	HX	−5.2	−10.3	−5.8	−8.5	−6.2	−7.4	−6.5	−7.6	−8.8	−5.9
**145**	HX	−5.3	−9.5	−6.1	−8.1	−6.9	−7.5	−7.2	−7.5	−8.3	−6.4
**146**	HX	−5.2	−9.5	−6.1	−8.2	−6.9	−7.5	−7.2	−7.5	−8.3	−6.5
**147**	HX	−5.0	−9.5	−5.8	−8.4	−6.8	−6.0	−6.2	−7.2	−7.2	−4.8
**148**	TePX	−6.4	−12.4	−6.8	−10.0	−8.1	−9.0	−8.3	−8.8	−10.1	−7.7
**149**	TePX	−6.1	−11.3	−6.9	−9.8	−8.2	−8.0	−7.5	−8.8	−10.2	−8.6
**150**	TePX	−6.1	−10.8	−6.5	−9.9	−7.7	−8.5	−7.4	−9.1	−9.9	−7.0
**151**	TePX	−6.0	−11.2	−6.6	−9.9	−8.4	−8.5	−8.0	−9.1	−9.9	−7.6
**152**	TePX	−6.0	−11.5	−6.7	−10.0	−9.2	−8.3	−8.8	−8.9	−9.2	−8.0
**153**	TePX	−5.8	−11.5	−6.6	−10.2	−8.2	−8.3	−8.3	−8.2	−9.6	−7.1
**154**	TePX	−6.3	−12.0	−7.4	−10.1	−8.0	−9.9	−8.5	−9.0	−10.2	−8.5
**155**	TePX	−5.8	−11.4	−7.3	−9.8	−8.2	−7.9	−7.7	−9.2	−10.3	−8.6
**156**	TePX	−6.4	−10.8	−6.8	−10.2	−8.1	−9.1	−8.3	−9.1	−9.7	−7.6
**157**	TePX	−5.8	−10.6	−6.3	−9.8	−8.3	−8.0	−8.6	−8.6	−9.8	−7.3
**158**	TePX	−5.9	−11.2	−6.8	−10.3	−8.4	−8.3	−7.9	−8.3	−10.4	−6.7
**159**	TePX	−6.1	−10.2	−6.9	−9.6	−8.4	−8.0	−7.6	−8.5	−10.0	−5.5
**160**	TePX	−5.7	−10.5	−6.5	−9.6	−8.4	−7.9	−8.5	−8.4	−9.7	−7.4
**161**	TePX	−5.8	−11.1	−6.5	−9.8	−7.6	−7.9	−7.5	−8.1	−10.1	−7.9
**162**	TePX	−5.7	−10.4	−6.2	−9.3	−7.6	−7.7	−7.3	−8.3	−9.7	−7.6
**163**	TePX	−6.0	−10.5	−6.7	−9.3	−8.2	−7.6	−7.9	−8.7	−9.7	−7.6
**164**	TePX	−6.4	−12.0	−6.8	−11.0	−8.6	−9.1	−8.0	−10.4	−10.5	−6.3
**166**	TePX	−6.6	−12.2	−6.7	−10.6	−9.9	−8.9	−8.6	−9.8	−10.7	−5.7
**167**	TePX	−6.4	−11.6	−6.6	−10.2	−7.8	−8.1	−8.5	−8.6	−9.9	−5.0
**168**	TePX	−6.5	−12.0	−6.5	−10.5	−7.9	−9.3	−8.6	−9.3	−10.3	−6.6
**169**	TePX	−6.3	−11.9	−6.8	−10.9	−8.8	−8.6	−7.7	−9.1	−11.0	−7.2
**170**	TePX	−6.7	−10.2	−6.9	−10.6	−8.4	−9.2	−8.7	−9.4	−10.7	−5.9
**171**	TePX	−6.2	−10.8	−6.8	−10.5	−8.1	−8.7	−7.8	−9.2	−10.2	−7.5
**172**	TePX	−6.7	−10.1	−6.8	−10.4	−8.7	−8.7	−7.8	−9.3	−10.0	−7.8
**173**	TePX	−6.4	−11.5	−6.5	−10.3	−7.9	−8.2	−8.4	−8.6	−9.9	−4.9
**174**	TePX	−6.6	−11.9	−6.5	−10.4	−7.9	−9.4	−8.5	−9.5	−10.3	−6.5
**175**	TePX	−6.3	−11.3	−6.6	−10.3	−7.8	−8.0	−8.4	−8.7	−9.8	−4.5
**176**	TePX	−6.0	−11.2	−6.5	−9.9	−7.4	−7.8	−8.0	−8.4	−10.6	−4.2
**177**	TePX	−6.0	−10.2	−6.5	−10.2	−7.8	−7.8	−7.8	−8.1	−9.2	−3.9
**178**	TePX	−5.9	−8.7	−6.3	−9.8	−7.6	−7.5	−7.5	−7.6	−9.3	−3.5
**179**	TePX	−6.0	−11.5	−7.0	−10.1	−8.2	−8.0	−8.0	−8.1	−11.2	−2.3
**180**	TePX	−6.1	−10.7	−6.5	−10.2	−7.8	−7.9	−7.8	−8.1	−9.4	−4.0
**181**	TePX	−6.0	−9.1	−7.0	−10.4	−7.9	−8.2	−8.1	−7.9	−10.3	−3.7
**182**	TePX	−6.5	−11.3	−6.9	−11.0	−7.9	−8.8	−8.1	−9.1	−10.9	−3.2
**183**	TePX	−6.2	−11.4	−6.6	−9.4	−8.1	−8.7	−8.1	−9.2	−9.9	−5.1
**184**	TePX	−6.4	−11.5	−6.5	−9.5	−8.1	−8.2	−8.3	−9.3	−9.3	−6.0
**185**	TePX	−6.2	−11.0	−6.8	−10.1	−8.1	−8.2	−7.5	−8.7	−11.0	−7.3
**186**	TePX	−6.3	−11.7	−6.8	−10.6	−8.4	−8.8	−8.2	−9.2	−10.3	−7.5
**187**	TePX	−6.4	−12.6	−6.5	−9.9	−8.3	−8.6	−7.8	−9.0	−10.3	−6.8
**188**	TePX	−6.5	−11.5	−6.7	−9.8	−8.0	−8.3	−8.1	−9.0	−10.9	−7.8
**189**	TePX	−6.2	−12.3	−7.2	−10.8	−8.7	−9.3	−8.0	−9.1	−10.8	−6.4
**190**	TePX	−6.5	−12.6	−7.3	−10.4	−8.8	−8.8	−8.5	−9.8	−10.8	−3.9
**191**	TePX	−6.1	−12.3	−6.7	−10.3	−8.2	−7.4	−7.9	−8.6	−10.2	−3.2
**192**	TePX	−6.6	−12.6	−7.0	−10.4	−8.1	−9.4	−8.2	−9.2	−10.1	−5.6
**193**	TePX	−6.2	−11.2	−6.7	−9.7	−8.1	−8.9	−7.9	−9.2	−9.9	−5.0
**194**	TePX	−6.6	−11.3	−7.0	−11.0	−9.0	−9.2	−8.4	−10.9	−11.1	−7.6
**195**	TePX	−6.4	−11.5	−6.9	−10.6	−9.3	−9.1	−8.4	−10.9	−11.0	−5.0
**196**	TePX	−6.7	−12.4	−6.4	−10.3	−8.0	−8.3	−7.9	−8.7	−11.2	−3.2
**197**	TePX	−6.6	−11.6	−6.8	−10.7	−8.7	−9.2	−8.1	−9.6	−11.0	−3.9
**198**	TePX	−6.4	−11.1	−6.8	−10.7	−9.0	−8.8	−8.0	−10.1	−11.8	−1.9
**199**	TePX	−7.1	−12.3	−7.0	−10.7	−8.3	−8.7	−7.9	−9.2	−10.5	−5.8
**200**	TePX	−6.8	−12.9	−7.4	−11.2	−9.1	−9.0	−8.5	−9.0	−10.7	−6.6
**201**	TePX	−6.4	−12.0	−6.9	−10.4	−8.6	−8.1	−8.7	−9.4	−11.1	−7.2
**202**	TePX	−6.3	−13.1	−7.2	−10.4	−8.8	−8.5	−8.9	−9.6	−10.5	−7.7
**203**	TePX	−6.3	−12.4	−7.2	−10.5	−9.0	−8.3	−8.7	−9.4	−10.2	−6.5
**204**	TePX	−6.3	−11.1	−7.1	−10.9	−8.3	−8.8	−8.9	−9.1	−10.8	−7.9
**205**	TePX	−6.2	−11.0	−7.1	−10.0	−7.9	−7.2	−8.0	−8.3	−10.5	−6.0
**206**	TePX	−6.4	−10.2	−7.1	−10.5	−7.9	−8.6	−8.2	−8.9	−10.7	−8.7
**207**	TePX	−6.4	−12.9	−7.3	−10.8	−8.9	−8.7	−9.1	−8.9	−10.4	−6.8
**208**	TePX	−6.1	−13.2	−6.9	−10.9	−8.7	−8.6	−9.0	−9.0	−10.8	−6.7
**209**	TePX	−6.5	−12.9	−7.2	−10.3	−9.0	−8.4	−7.9	−9.4	−10.3	−6.7
**210**	TePX	−6.5	−11.7	−7.6	−10.5	−8.7	−8.7	−8.4	−9.0	−10.4	−7.1
**211**	TePX	−6.1	−11.7	−6.7	−11.2	−9.2	−8.8	−9.2	−9.6	−11.0	−6.7
**212**	TePX	−7.5	−11.9	−7.8	−11.3	−8.6	−9.0	−8.6	−9.3	−10.8	−5.5
**213**	TePX	−7.7	−12.9	−7.4	−11.6	−9.1	−9.4	−9.1	−9.5	−12.2	−4.7
**214**	TePX	−8.0	−9.0	**−8.9**	−11.7	−8.9	−9.4	−9.0	−9.6	−11.8	−3.2
**215**	TePX	−7.7	**−13.7**	−8.3	−11.5	**−9.9**	−9.5	−8.3	−9.7	−12.3	−4.0
**216**	TePX	−7.2	−11.2	−8.1	−11.3	10.0	−9.9	−9.2	−10.6	−12.1	−5.8
**217**	TePX	−7.1	−11.5	−7.8	−11.1	−9.7	−9.9	−8.8	−11.1	−11.7	−6.9
**218**	TePX	−6.8	−13.4	−7.7	−10.4	−9.0	−9.1	−8.6	−10.7	−12.2	1.8
**219**	TePX	−7.2	−13.5	−8.1	−11.6	−9.1	−10.1	−9.0	−10.3	−11.8	−3.6
**220**	TePX	−7.4	−13.2	−7.9	−12.3	−9.6	−8.8	**−10.0**	−9.9	−12.5	−3.7
**221**	TePX	−7.3	−12.1	−8.5	−11.5	−9.0	−8.9	−8.3	−9.3	−10.5	−3.0
**222**	TePX	−6.3	−12.9	−7.0	−10.7	−8.8	−8.6	−8.3	−9.7	−12.0	−5.8
**223**	TePX	−7.3	**−13.7**	−7.3	−10.2	−8.6	−9.3	−9.1	−9.2	−10.2	−5.5
**224**	TePX	−6.5	−11.2	−7.1	−10.5	−8.6	−8.6	−8.1	−9.4	−12.0	−5.5
**225**	TePX	−7.1	−12.8	−7.2	−11.1	−8.8	**−10.2**	−8.6	−10.0	−10.5	−5.7
**226**	TePX	−6.7	−12.5	−7.2	−10.8	−9.1	−9.4	−8.9	−10.2	−12.1	−2.6
**227**	TePX	−6.8	−13.6	−7.4	−11.4	−9.3	−8.4	−8.8	−10.0	−12.2	−5.4
**228**	TePX	−6.6	−13.3	−6.9	−10.5	−8.9	−7.9	−9.2	−9.8	−11.8	−2.9
**229**	TePX	−6.8	−11.3	−7.0	−11.4	−8.1	−9.2	−8.8	−9.2	−10.7	−6.4
**230**	TePX	−6.4	−13.3	−7.1	−10.6	−8.7	−8.7	−8.0	−9.3	−11.6	−3.1
**231**	TePX	−6.9	−13.4	−7.5	−11.7	−8.9	−8.9	−8.3	−9.0	−11.1	−5.9
**232**	TePX	−7.2	−9.3	−7.8	−10.6	−8.7	−9.8	−8.2	−10.1	−11.3	−7.4
**233**	TePX	−6.6	−13.7	−7.4	−10.8	−9.0	−8.5	−8.7	−9.3	−10.8	−5.4
**234**	TePX	−6.1	−11.9	−6.9	−10.3	−8.6	−7.2	−7.9	−9.0	−11.6	−0.9
**235**	TePX	−6.9	−10.8	−7.1	−9.8	−8.4	−9.1	−8.1	−9.3	−10.0	−5.8
**236**	TePX	−6.4	−13.0	−7.1	−11.3	−8.8	−8.7	−8.6	−9.2	−10.6	−5.3
**237**	TePX	−6.9	−12.1	−7.0	−10.6	−8.2	−8.8	−8.3	−9.8	−10.3	−5.9
**238**	TePX	−6.7	−12.0	−7.5	−11.2	−8.5	−8.3	−8.3	−10.1	−11.8	−6.3
**239**	TePX	−6.7	−12.1	−6.9	−10.5	−8.1	−8.8	−8.1	−9.2	−10.7	−6.6
**240**	TePX	−7.2	−13.2	−7.5	−11.1	−8.0	−9.4	−8.5	−9.6	−10.4	−4.5
**241**	TePX	−7.4	−13.0	−7.5	−11.6	−8.7	−9.9	−8.2	−10.3	−11.7	−5.2
**242**	TePX	−6.9	−12.8	−6.9	−10.8	−8.9	−8.9	−8.3	−10.4	−12.0	−4.3
**243**	TePX	−6.6	−13.2	−7.0	−11.1	−8.9	−8.3	−8.0	−9.5	−11.1	−1.8
**244**	TePX	−6.8	−13.3	−7.7	−11.2	−9.1	−8.5	−8.9	−9.9	−11.6	−5.1
**245**	TePX	−6.6	−11.1	−7.6	−11.1	−9.2	−8.3	−7.7	−10.1	−11.3	−6.6
**246**	TePX	−6.4	−12.3	−7.2	−10.9	−9.0	−8.8	−8.0	−9.6	−10.6	−3.3
**247**	TePX	−6.3	−12.5	−7.5	−9.9	−8.6	−8.8	−8.0	−9.9	−11.4	−1.7
**248**	TePX	−6.7	−13.2	−7.4	−10.5	−8.9	−7.6	−8.5	−8.9	−11.8	−5.2
**249**	TePX	−6.5	−13.2	−7.2	−10.7	−9.2	−8.2	−8.0	−9.1	−10.9	−1.3
**250**	TePX	−6.4	−12.1	−6.7	−10.7	−8.9	−8.1	−8.5	−9.5	−10.0	−3.6
**251**	TePX	−6.1	−11.4	−6.8	−10.6	−8.7	−7.8	−7.5	−10.1	−10.0	−2.8
**252**	TePX	−5.9	−10.8	−6.2	−10.2	−7.9	−7.0	−7.6	−8.0	−10.6	−1.0
**253**	TePX	−7.6	−11.2	−7.4	−12.0	−9.2	−10.0	−8.7	−10.1	−12.6	−5.1
**254**	TePX	−7.2	−9.5	−8.1	**−12.4**	−9.3	−9.1	−8.9	−10.1	−11.4	−5.8
**255**	TePX	−7.0	−12.6	−7.0	−10.7	−8.5	−9.2	−8.0	−9.0	−12.8	−4.6
**256**	TePX	−7.3	−10.5	−7.7	−11.3	−8.6	−9.8	−8.5	**−11.4**	**−13.9**	−3.2
**257**	TePX	−6.8	−11.3	−7.4	−10.8	−8.9	−8.1	−8.1	−9.9	−12.3	−3.1
**258**	TePX	−7.2	−10.6	−8.1	−10.8	−9.2	−9.2	−8.2	−8.6	−10.3	−1.7
**259**	TePX	−6.5	−10.4	−6.7	−9.8	−8.7	−8.9	−7.6	−9.2	−10.4	−4.1
**260**	PePX	−6.3	−11.2	−6.7	−10.9	−8.6	−8.5	−7.6	−9.2	−10.4	−5.6
**261**	PePX	−6.8	−12.0	−7.4	−10.4	−8.4	−8.9	−7.8	−8.8	−10.5	−7.4
**262**	PePX	−6.2	−11.1	−7.3	−10.9	−8.9	−8.8	−7.6	−9.5	−9.6	−6.3
**263**	PePX	−6.3	−11.9	−7.1	−9.8	−8.6	−8.7	−7.8	−9.0	−10.5	−4.7
**264**	Dim	−7.3	−13.4	−8.2	−11.7	−8.5	−9.0	−8.9	−10.3	−12.7	−7.9
**265**	Dim	−7.6	−9.0	−7.6	−10.3	−8.9	−9.3	−8.6	−9.3	−11.4	−1.7
**266**	Dim	**−8.4**	−11.7	−8.0	−10.6	10.2	−9.8	−8.9	−10.8	−11.5	1.2
**267**	Dim	−6.4	−2.7	−7.1	−5.7	−8.9	−8.4	−8.1	−9.2	−9.8	30.7
**268**	XD	−6.9	−11.3	−7.9	−11.8	−9.5	−8.9	−8.1	−9.7	−10.5	−0.7
**269**	XD	−6.8	−11.2	−7.5	−9.8	−9.3	−8.7	−7.4	−8.5	−10.3	−0.9
**270**	XD	−7.1	−10.2	−7.9	−9.8	−9.1	−8.4	−7.5	−8.4	−10.6	−2.1
**271**	XD	−6.9	−7.4	−7.9	−10.0	−9.0	−7.9	−7.5	−7.8	−10.6	−1.4
**272**	XD	−7.2	−8.7	−8.0	−10.2	−8.9	−7.6	−8.0	−9.2	−10.5	−2.3
**273**	CCI ^b^	−9.4									
**274**	CCI ^b^		−11.1								
**275**	CCI ^b^			−7.0							
**276**	CCI ^b^				−8.7						
**277**	CCI ^b^					−7.9					
**278**	CCI ^b^						−7.2				
**279**	CCI ^b^							−7.9			
**280**	CCI ^b^								−9.7		
**281**	CCI ^b^									−10.4	
**282**	CCI ^b^										−7.1

^a^ Mean of the AutoDock Vina score for 10 replicates; ^b^ Co-crystalized inhibitors for each enzyme: **273** = *N*-[(1*R*,2*R*)-2-carbamimidamido-2,3-dihydro-1*H*-inden-1-yl]-*N*′-(4-chloro-3-fluorophenyl)ethanediamide; **274** = 5-{[3,5-diethyl-1-(2-hydroxyethyl)-1*H*-pyrazol-4-yl]oxy}benzene-1,3-dicarbonitrile; **275** = guanosine-2′-monophosphate; **276** = 2-(2,4-difluorophenyl)-1,3-di(1*H*-1,2,4-triazol-1-yl)propan-2-ol; **277** = 2-hydroxymethyl-5-(4-nitro-phenoxy)-tetrahydro-furan-3,4-diol; **278** = β-l-fucose; **279** = 2-heptyl-4-hydroxyquinoline *N*-oxide; **280** = [cyclohexylethyl]-[[[[4-[2-methyl-1-imidazolyl-butyl]phenyl]acetyl]-seryl]-lysinyl]-amine; **281** = (1-methyl-1*H*-imidazol-2-yl)-(3-methyl-4-{3-[(pyridin-3-ylmethyl)-amino]-propoxy}-benzofuran-2-yl)-methanone; **282** = (1*S*)-*N*,4-dimethyl-*N*-(4-methylpent-3-enyl)cyclohex-3-enaminium.

**Figure 12:**
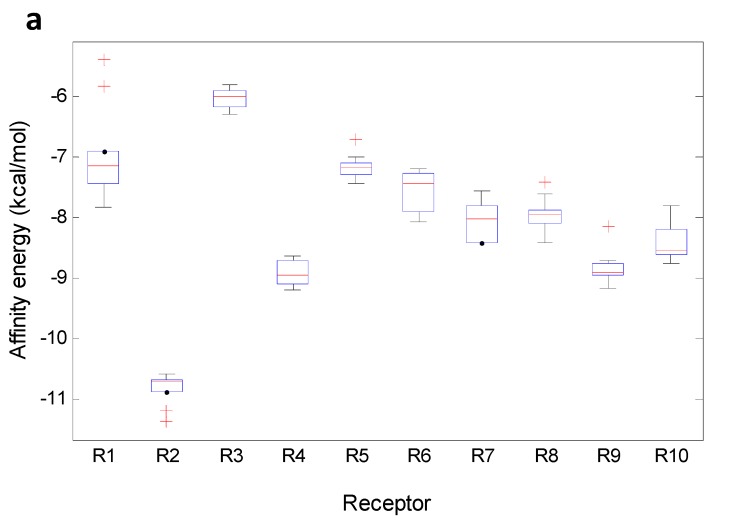

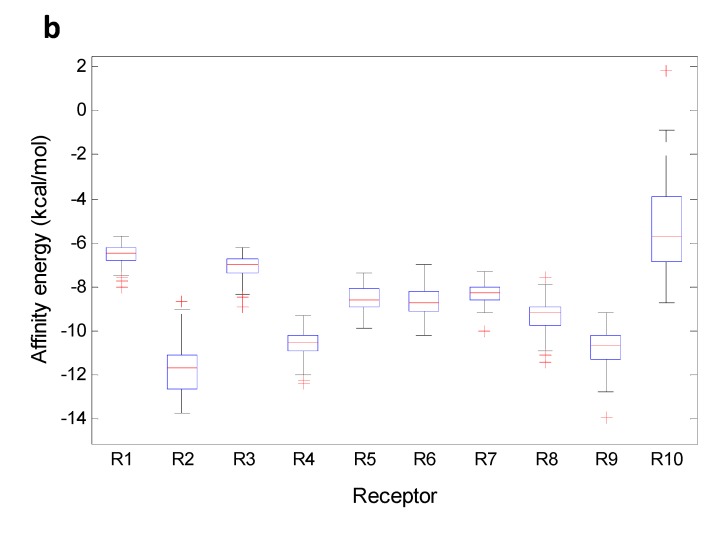
Boxplot for affinity values of the selected xanthone-types. (**a**) MX; (**b**) TePX.

Non-normal distribution of the data was expected due to significant differences in the structural interactions between the analyzed xanthones and the targets. Therefore, boxplots were constructed for the data matrix considering the 10 previously defined xanthone groups. Figure 12 shows boxplots for MX and TePX. The boxplots for the other xanthone types are presented as Supplementary Material. Almost normal distribution of the data was found to R3, R4, R6, R7 and R10 when MX acted as ligand. For its part, data of R1 for MX were found to be markedly disperse including some atypical values higher than the corresponding median (Figure 12a). Minimum affinity energy for ligand—receptor complexes were found for MX—R2, meanwhile MX demonstrated to be inadequate inhibitors for R3.

The general performance of the docking energy values for the studied receptors was firstly analyzed by Pearson’s correlation as shown in Table 3. R10 demonstrated to have a slightly negative correlation with R1, R3–R9. Highest positive correlations were found for R9 with R3, R4 and R8; in similar way R8 showed high positive correlation with R4. Crude means and relative standard deviation percentages (%RSD) for whole affinity data are also presented in Table 3. High variance in data was observed from corresponding RSD (Table 3). A Tukey test was also performed exhibiting similar behavior between R5, R6 and R7 and between R4 and R9. The last one was in agreement with that defined by Pearson’s correlation.

Atypical distribution of data for R2—MX affinity was clearly observed. For that enzyme, the median was almost on the 75th percentile and two low energy outliers were noticed. Outliers lower than the median in terms of affinity energy means stronger interaction and potentially better inhibition of the target enzyme. In this case, **3** and **8** were outliers for R2. R4 and R9 similarly interacted with MX which is in agreement with that observed from whole data (Table 3). On the other hand, TePX exhibited more variable data than those for MX along R1–R10 (Figure 12b). From these, affinity of TePX for R2 and R10 showed the highest inter-group variability. Close behavior in the affinity values distribution were observed for TePX with R5, R6 and R7. It can be seen a similar performance of TePX on all tested enzymes which could be related with previously found observations. DX resulted to be able to form stable complexes with R2, while complexes with R1 and R3 were not energetically favored compared with the others. Similar performance was noticed for TrX, although variance within data was significantly different. Complexes between R6, R7 or R10 and TeX showed a wide range of affinity values. PeX showed also high variability among data with R6, R9 and R10. In the case of HX, an unsymmetrical data distribution was clearly noticed in affinity values with R2, R5 and R6. Low affinity of XD for R10 and high variance of those for R2 were found. Finally, R5— and R10—Dim complex formation can be proposed to be non-spontaneous processes due to their lack of affinity (characterized by positive affinity values). A holistic view of the data (Table 3 and Figures in the Supplementary Material) allowed establishing partial groupings amongst MX and DX, TrX, TeX and HX, and TePX, PePX and XD according to mean affinity and their variance across R1–R10.

**Table 3:** Mean docking scores for each target enzyme and their Pearson’s correlation.

ID	Mean Affinity ^a^ (kcal/mol)	RSD ^b^ (%)	Pearson’s Correlation ^c^								
			R1	R2	R3	R4	R5	R6	R7	R8	R9
R1	−6.1	11.4 ^A^									
								
R2	−10.8	11.6 ^F^	0.471								
			(0.000)								
R3	−6.6	9.4 ^B^	0.700	0.445							
			(0.000)	(0.000)							
R4	−9.7	9.9 ^E^	0.627	0.708	0.768						
			(0.000)	(0.000)	(0.000)						
R5	−7.7	22.2 ^C^	0.095	0.203	0.194	0.244					
			(0.117)	(0.001)	(0.001)	(0.000)					
R6	−8.1	9.8 ^C^	0.641	0.482	0.702	0.710	0.134				
			(0.000)	(0.000)	(0.000)	(0.000)	(0.027)				
R7	−7.9	7.2 ^C^	0.568	0.481	0.587	0.608	0.117	0.630			
			(0.000)	(0.000)	(0.000)	(0.000)	(0.055)	(0.000)			
R8	−8.5	10.7 ^D^	0.669	0.595	0.767	0.818	0.182	0.770	0.588		
			(0.000)	(0.000)	(0.000)	(0.000)	(0.003)	(0.000)	(0.000)		
R9	−9.6	12.7 ^E^	0.695	0.614	0.802	0.842	0.226	0.696	0.549	0.871	
			(0.000)	(0.000)	(0.000)	(0.000)	(0.000)	(0.000)	(0.000)	(0.000)	
R10	−6.5	47.3 ^B^	−0.312	0.041	−0.415	−0.199	−0.092	−0.243	−0.151	−0.412	−0.446
			(0.000)	(0.501)	(0.000)	(0.001)	(0.131)	(0.000)	(0.013)	(0.000)	(0.000)

^a^ Mean value from 10 replicates; ^b^ Capital letters in this column represent different group by Tukey’s test; ^c^ *p*-values are shown in parenthesis.

**Figure 13:**
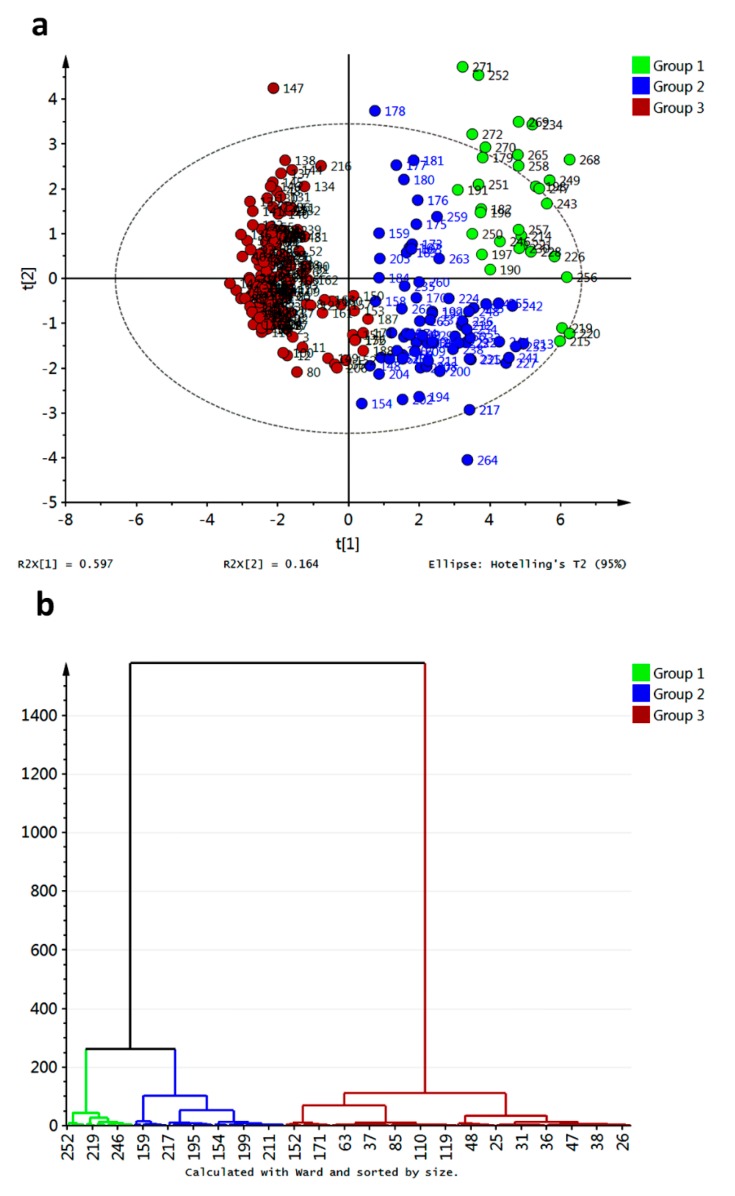
Unsupervised multivariate analyses from affinity values for docking of xanthones with target enzymes. (**a**) PCA score plot; (**b**) HCA dendrogram.

In order to go into detail about the relationship between affinity energies and xanthone structural features, multivariate analyses were employed. Principal component analysis (PCA) and hierarchical clustering analysis (HCA) were performed on the data matrix and results are shown in Figure 13.

Compounds **218**, **266** and **267** resulted as outliers of the PCA model of the raw data and should be further removed to improve the statistical significance of the score plot. Partial grouping of xanthone types was observed in the PC1–PC2 score plot when raw data were employed (data not shown). After removal of the outliers, a similar distribution along the PC1–PC2 score plot was found, although three clusters can be defined based on HCA (Figure 13). Clustering in the data matrix proved statistically significant differences among xanthone performances as potential inhibitors of R1–R10. Clusters 1 and 2 (green and blue marks, respectively, in Figure 13a) resulted in the most disperse group, with apparent great number of links (Figure 13b). For its part, cluster 3 (red marks) was the group with the minor dispersion along PC1–PC2 score space (Figure 13a). Nevertheless, this cluster also consisted of high number of links (Figure 13b). This cluster was constituted by MX, DX, TrX, TeX, PeX and HX. In-depth analysis let to establish that cluster 3 is subdivided into two groups: one subgroup mainly consisted of PeX, HX and 38% of total TeX together with three DX-related compounds (**26**, **28**, **30**) which are permethylated ones. Compounds **161** and **162** were atypically included as part of cluster 3 indicating significant differential interaction of these structures with the tested enzymes regarding the rest of TePX. These bearing a 1,1-dimethylallyl chain at C4 and OH groups at C2, C5, and C6. Cluster 1 was mainly constituted of XD and 52.3% of TePX while cluster 2 was formed by PePX and 45.9% of TePX. TePX bearing one prenyl chain as well as monofuran- and monopyranxanthones were found in cluster 2. For its part, cluster 1 contained synthetic derivatives of bisprenylated xanthones and xanthones with prenyl together with furan- and/or pyran- units as structural features. By comparison of the minimum energy values into each cluster, the first one can be established as that with the best inhibitory potential against R2–R9. At the same time, cluster 3 resulted in the poorest inhibitor xanthones for R2–R9, although these structures were demonstrated to form the strongest complexes with R10. As mentioned above, none of the analyzed structures was a good inhibitor for R1 based on the corresponding affinities.

**Figure 14:**
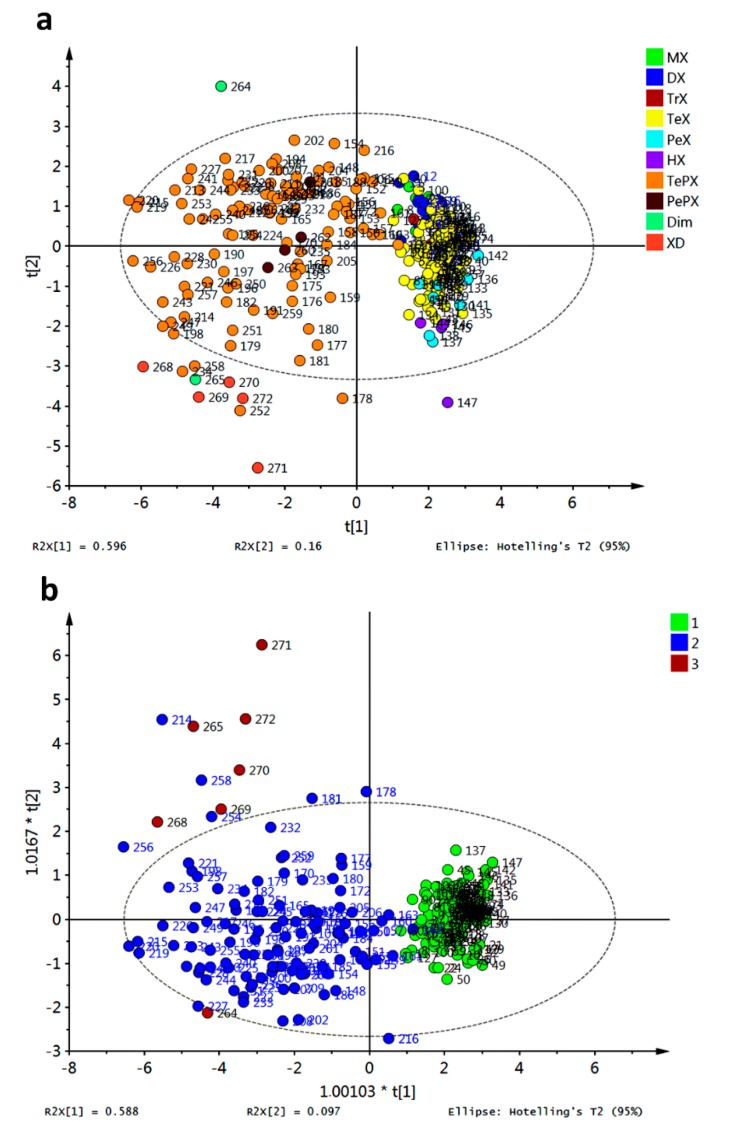
Supervised multivariate analyses from affinity values for docking of xanthones with target enzymes. (**a**) PLS-DA score plot; (**b**) OPLS-DA score plot. Group 1: non-prenylated; Group 2: prenylated; Group 3: Dim and XD.

Partial Least Square—Discriminant Analysis (PLS-DA) was attempted on the data matrix after exclusion of the abovementioned outliers and the resulting score plot is shown in Figure 14a. As observed, dispersion of data across *X*-*Y* scores was evident. However, grouping of all non-prenylated compounds on positive values for *X*-score was found (MX, DX, TrX, TeX, PeX, and HX). Therefore, Orthogonal Partial Least Square—Discriminant Analysis (OPLS-DA) was subsequently performed employing prenylation as classification criterion (Figure 14b). Good discrimination of the affinity energy dataset based on prenylation (structure) criterion was obtained, demonstrating meaningful relationship between affinities of xanthones for R1–R10 and the presence of prenyl and biosynthetically prenyl-related groups in the xanthone structure. At this regard, observed clustering in HCA was completely in concordance with the OPLS-DA results. High number of outliers for PLS-DA and OPLS-DA were observed, indicating an exceeding variance among data, and thus model compliance was not fully achieved. That information was in agreement with previous findings from boxplots and PCA.

### 2.2. Molecular Docking and Chemical Interactions

**Figure 15:**
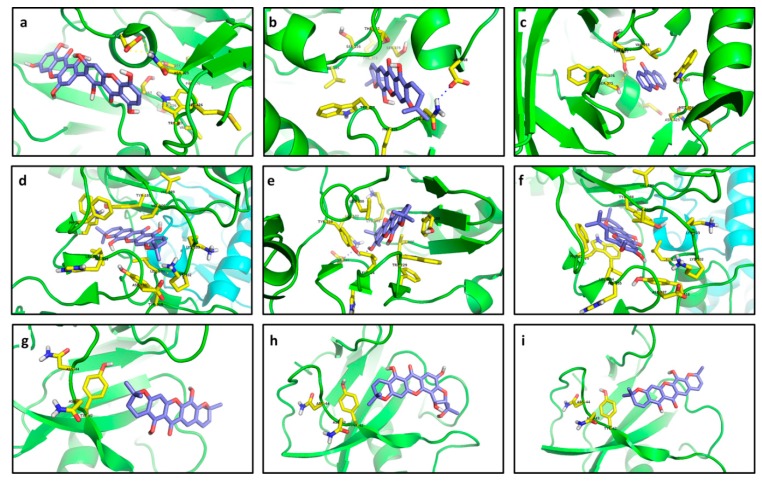

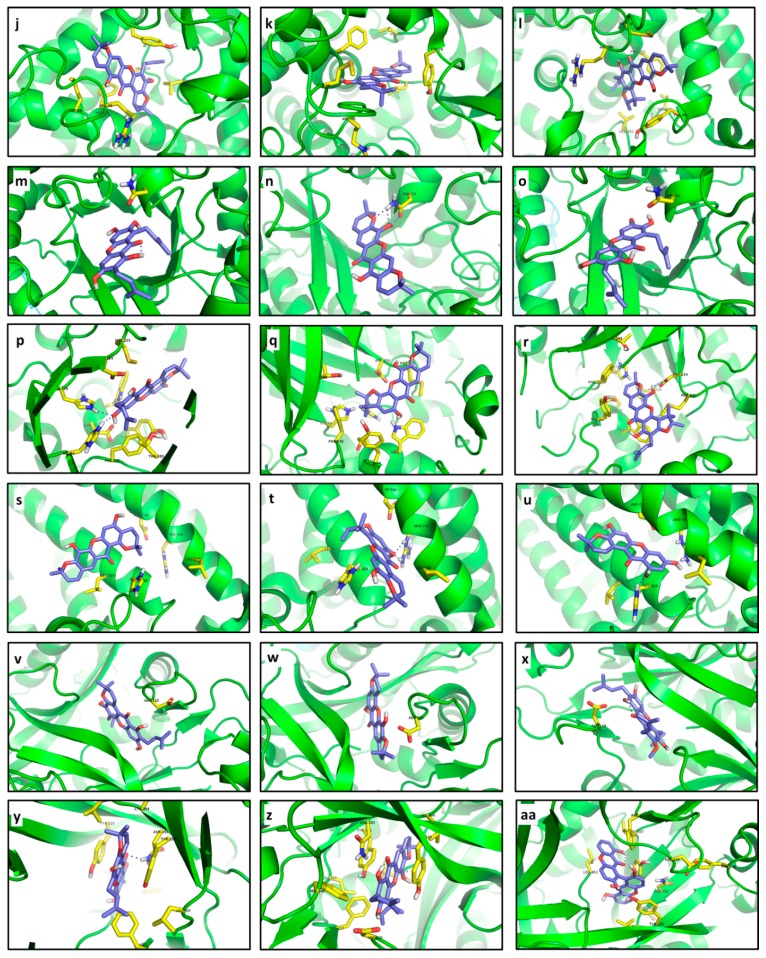

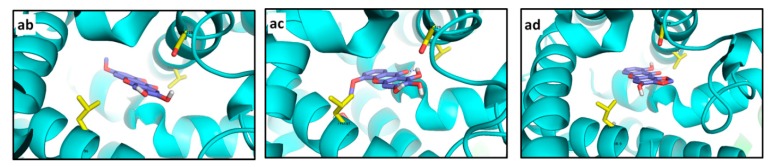
Best docked poses for tested xanthones. (**a**) **266**—R1; (**b**) **214**—R1; (**c**) **1**—R1; (**d**) **215**—R2; (**e**) **223**—R2; (**f**) **233**—R2; (**g**) **214**—R3; (**h**) **221**—R3; (**i**) **215**—R3; (**j**) **254**—R4; (**k**) **220**—R4; (**l**) **253**—R4; (**m**) **166**—R5; (**n**) **215**—R5; (**o**) **217**—R5; (**p**) **225**—R6; (**q**) **219**—R6; (**r**) **253**—R6; (**s**) **220**—R7; (**t**) **228**—R7; (**u**) **211**—R7; (**v**) **256**—R8; (**w**) **217**—R8; (**x**) **195**—R8; (**y**) **256**—R9; (**z**) **255**—R9; (**aa**) **264**—R9; (**ab**) **116**—R10; (**ac**) **100**—R10; (**ad**) **80**—R10.

Compounds **266** and **214** were characterized as the best inhibitors for R1 according to docking results (−8.4 and −8.0 kcal/mol, respectively) in spite of their poorer interactions than that with CCI **273** (−9.4 kcal/mol). Compound **266** did not show any polar contact with residues in the binding site of R1; interactions with residues near the binding site were estimated as probably exclusively controlled by steric hindrance (Figure 15a). Only a relative proximity of 5-OH to Asp368 and Asn425 (2.9 and 2.7 Å aprox.) could be observed. In contrast, the aliphatic chain in **273** let it to introduce into the pocket and cling to the residue Asp368 of R1 by hydrogen bonding (Figure 15c). Although **214** established only non-polar interactions with R1, it was clearly able to perfectly introduce into the pocket conformed by Ser375, Thr257, Val255 and Trp427 (Figure 15b). In the case of R2, compounds **215**, **223**, **233** and **227** exhibited the strongest affinities, ranging from −13.72 to −13.61 kcal/mol. Such compounds established stronger interactions with R2 than that with CCI **274** (Table 3). Coplanarity of **215** rings gave a stable complex with π-π interactions between its B ring and Tyr188 (Figure 15d). No polar contacts were defined for **215**—R2 complex but probable non-polar interactions with Leu100, Lys102, Lys103 and Tyr318, together with medium proximity of Trp229 could be stabilizing the complex. Hydrogen bonding with Tyr318 and π-π stacking with Tyr188 were the key interactions in the formation of the **223**—R2 complex (Figure 15e). Only non-polar interactions among R2 and **233** were predicted, including a π-stacking with Trp229. Inferred non-polar contacts involved several residues such as Val106 and Tyr188 (Figure 15f). A suitable distance between Tyr318 and 8-OH (3.1 Å aprox.) allow us to infer the probable formation of hydrogen bonds in the **233**—R2 complex. CCI **274** showed π interaction with Tyr188.

While affinity differences were almost absent, structural interactions were totally different among these compounds, indicating that the presence of the two pyran rings on **215** let it interact more efficiently with the binding site residues. At the same time, **233** exhibited a lesser number of interactions than **215**, but the presence of π-stacking can contribute to high stabilization of the complex, explaining thus the similar affinities. A greater number of the tested xanthones were able to establish stronger interactions with R3 than with CCI **275**. Compounds **214**, **221** and **215** displayed the lowest affinity energy and could be then proposed as possible naturally-occurring inhibitors of R3 (−8.92 to −8.33 kcal/mol). Nevertheless, **214** did not establish interactions with the selected flexible residues (distance upper than 5.7 Å; Figure 15g). This behavior was retained in the other compounds that demonstrated strong docking with R3. Structural dissimilarity between **214** and **215** consisted of a double bond at C3-C4. However, affinity differences were noticed, demonstrating the importance of the C3-C4 double bond in stabilization of the complex with R3. On the other hand, considerably strong complexes were obtained between **254**, **220** and **253**, and R4, with affinity values ranging from −12.4 and −12.0 kcal/mol. Despite their lowest affinities, these receptor–ligand complexes were restricted to non-polar interactions (Figure 15j–l).

In the case of **254**, π interactions amongst the prenyl unit and Tyr76 were detected (Figure 15j). Moreover, this prenyl unit could be supporting non-polar contact with Leu321, indicating that the existence of a prenyl unit in the ligand can be a key structural feature in complex establishment. The formation of the **220**–R4 complex included non-polar contacts between a pyran ring of **220** with Val434 and Leu321 as well as between the other pyran ring with Met99 and Arg96 (calculated distances in the 3.4–3.8 Å range; Figure 15k). **220** exhibited greater interactions number than those of **254** (Figure 15j,l), but affinities behaved in opposite form. Thus, the increase of hydrophobic contacts does not strictly result in an increase of stabilization of the receptor‒ligand complex.

Compounds **166** and **215** gave the same affinity to R5 despite their structural differences (Table 3). Only a possible interaction of 7-OH in **166** with Asn74 could be inferred from the distance between them, although Pymol was not able to determine any polar contact there (Figure 15m). In contrast, **215** undoubtedly showed three hydrogen bonds with Asn74 (Figure 15n). Although **166** and **255** were not structurally related, their complexes with R5 resulted in exactly the same energy. Regarding R6, its interaction with the tested ligands was significantly stronger than that calculated for CCI **278** (Table 2), being **225** the compound with the most important interaction. Hydrogen bonds of 9-OH with His34 and His128 were clearly determined in the **225**—R6 complex (Figure 15p). Besides, a non-polar interaction with Trp67 as well as a polar interaction with Asp224 can be proposed. Unlike **225**, no polar contacts were established by **219** with R6. Nonetheless, relative proximity (distances lesser than 4.0 Å) of **219** and several residues of the binding site pocket of R6, including Asp224, Trp67, Phe290, Tyr64, Met225, His34, and His128, let us infer a marked stabilization of the predicted complex (Figure 15q). Compound **253** were also seen to exhibit strong interactions with R6. In the best docked pose of **253**, an H-bond with Asp224 was clearly formed together with probable interactions with Met225, His34 and His128 (Figure 15r). The presence of a prenyl unit on C2 in **253** seems to cause steric hindrance and therefore key interactions were performed by the pyran ring, while the furan ring was significantly more important in **219**—R6 complex formation.

Particularly, docking of R7 resulted in completely different molecular interaction pathways. Polar contacts were not detected for the **220**—R7 complex, whose main interaction resulted from nearness to Leu325 (3.5 Å) and His328 (3.1 Å). Particularly, despite the strong predicted interaction (very low affinity value), the best pose for **220** at R7 was adopted out of the pocket (Figure 15s), making **220** a non-competitive inhibitor. In contrast, **228** exhibited a double H-bond with Arg742 and was exactly placed into the R7 pocket (Figure 15t). Additionally, the 2.8 Å distance between His328 and the oxygen atom in the γ-pyrone-unit allows us to propose a polar interaction there. Similarly, H-bonds with His328 and Arg742 were evidently observed for **211** (Figure 15u). Compound **228** possesses a 1,1-dimethylallyl group at C4 that is different from **211** but their relative complex strength was the same. Thereof, no conclusions can be outlined about the effect of the 1,1-dimethylallyl group. Like R5, docking calculations for R8 were carried out with only one flexible residue as determined by the AutoDock Vina plugin. Xanthones **256**, **217** and **195** exhibited the best affinities for R8 (−11.4 to −10.9 kcal/mol; Figure 15v–x), although the difference with respect to CCI **280** was quite low (9.7 kcal/mol) compared with those for other enzymes. These xanthones were located near to Asp110 at distances ranging between 3.1–3.4 Å. The best inhibitor for R9 was also **256**. A polar contact with Asn392 as well as non-polar interactions with Tyr225, Leu337, Phe117, and Asp110 were found in **256**—R9 (Figure 15y). π-stacking with Tyr354 was also evident in the **256**—R9 complex. Contrarily, no polar contact was determined for **255** (Figure 15z). Nevertheless, π-stacking with Tyr225 and interaction with Asn392, Asp110, Tyr354, and Phe117 could be proposed due to distances lesser than 4.0 Å between R9 and **255**.

In the case of **264**, double hydrogen bonding was selectively formed with Tyr225 (Figure 15aa). Moreover, Asn392 and Tyr354 appeared to be very close to this ligand (3.7 and 3.2 Å, respectively), so that interactions with these residues can be then established. Enzyme R10 markedly interacted with **116**, **100** and **80** in decreasing strength. None of these xanthones established polar contacts with binding site pocket residues (Figure 15ab–ad). However, non-polar interactions with Val222 for **116**, with Leu97 and Thr96 for **100**, and with Leu97 and Val222 for **80** can be proposed. A distance of 4.0 Å between 3-OH at **100** and Ser229 at R10 let us infer a weak polar interaction between them. Great structural differences between the analyzed ligands made it impossible to drawn particular conclusions about pharmacophore groups.

**Figure 16:**
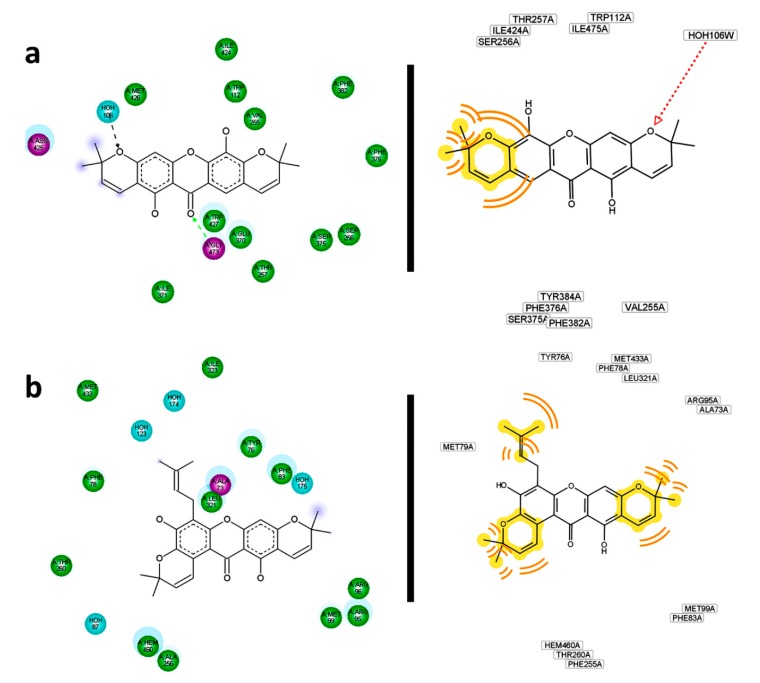

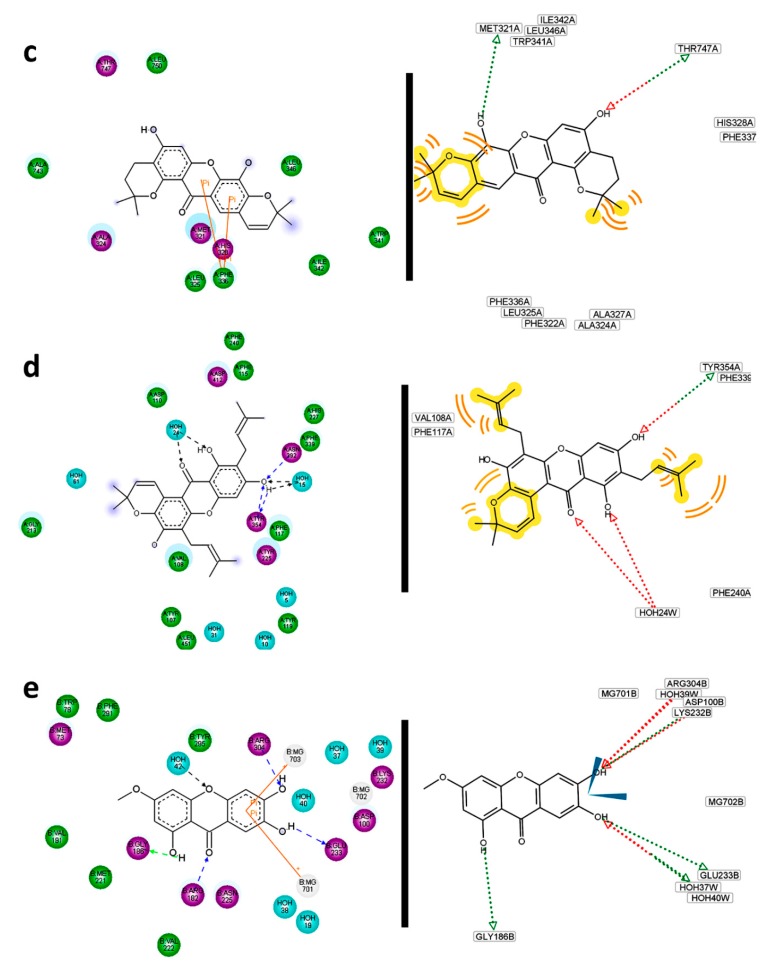
Residual interaction maps for selected structures constructed by Discovery Studio (left) and Ligand Scout (right). (**a**) **215**—R2; (**b**) **254**—R4; (**c**) **220**—R7; (**d**) **256**—R8; (**e**) **116**—R10.

Further analysis regarding residual interactions in ligand—receptor complexes was carried out using the Discovery Studio and LigandScout softwares, in order to get detailed information on structural requirements providing stronger ligand—receptor interactions. Residual interaction maps were then obtained as shown in Figure 16. In depth, these analyses did provide other important ligand—receptor interactions as additional support to explain the observed behaviors. The most important interactions found are described as follows: a polar contact between Ser365 and 1-OH in the **266**—R1 complex can be proposed. Moreover, interactions of 5-OH with Asp457 and Asp368 were confirmed. No compliance with the most important residues reported as interacting with CCI **273** was found. For its part, **215** could establish a polar contact with Gly473 of R2. Simultaneously, hydrophobic interactions from the pyran ring could be generated (Figure 16a). Observed and predicted interacting residues were not in agreement with those reported in the **274**—R2 complex. In the case of **214**—R3 complex, residual interaction maps were employed to infer its strength. Hydrophobic interactions of methyl groups were evidently noticed. Likewise, a π-π interaction between the benzo-γ-pyrone and Arg77 was delimited as the key contact. By comparing the reported R3 binding site residues for **275**, several similarities can be indicated, including Tyr42, Tyr45, Glu46 and Glu58, besides Asn98 and Tyr38 which could define specific polar contacts with **214**. In addition, hydrophobic contacts of the pyran rings and prenyl chain of **254** were key features when it interacted with R4 (Figure 16b). Tyr76 and Leu321 were confirmed as important residues in the interaction and stabilization of the **254**—R4 complex. These observations were in agreement with those for CCI **276**.

Residual interaction maps were an essential tool for extracting and understanding the formation of complexes with R5 where no flexible residues were employed. For **166**, significant interactions with Asn217, Trp180, Leu31, Asn174 and Glu294 can be proposed. For the **225**—R6 complex, hydrogen bonds mentioned above were confirmed.

Simultaneously, interactions with Phe32, Phe290, Trp222, His129, Arg254, Glu266, Phe59 and Pro188 were defined by its residual interactions map. Some of them have been previously reported into the binding site interactions with **278**. The importance of residual interaction maps was also demonstrated for the **220**—R7 complex. For this, possible interactions of **220** with Met321, Thr747, Phe336 were defined (Figure 16c). Interactions with the binding site pocket were more efficiently described than Pymol observations with residual interaction maps in the case of the **256**—R8 complex. In fact, polar contacts could be expected with Tyr354 and eventually with Asn392. In this way, hydrophobic interactions to **256** could be also predicted by Val108, Phe117, Phe115, and likely Tyr225, Asp412 and Phe240 (Figure 16d). Many of these were previously reported as residues of the R8 pocket. For **256**—R9 complex, most of interacting residues described above were confirmed by residual interaction map analysis. However, many other important contacts can be observed, including polar contacts with His227, Tyr354 and Tyr335, in addition to hydrophobic contacts mediated by Tyr107 and Phe240. As expected most of the reported R9 binding site residues agreed with those found by interaction with **256** since it was able to perfectly adjust to this pocket. Compound **256** was demonstrated to display the best interaction with R8 as well as with R9. This fact can be rationalized because R8 and R9 are different crystalized structures of the same enzyme. For the first one, structural analysis was obtained on co-crystalizing with a peptidic inhibitor, while for the last one, the crystallographic data were obtained from an enzyme–non-peptidic inhibitor complex. The sequences in R8 and R9 are exactly same, therefore differential interactions of enzymes R8 and R9 with ligands can be attributed to slight differences on the tertiary structure occurring during the crystallization process. The location of the inhibitor in the binding site can induce changes in the tridimensional conformation of the pocket and hence the availability of particular residues for interaction with ligands would be also changed. On the other hand, residual interactions of **116** with R10 were effectively demonstrated. In depth, polar contacts with Arg304, Glu233 and Gly186 can undoubtedly be proposed (Figure 16e).

### 2.3. Approach to Relationship Docking Affinity–Antifungal Activity 

The structural simplicity of MX, DX and even TrX can be used to evaluate an affinity—activity relationship model. Hence, two previously reported antifungal activity datasets were employed in the present research as a discriminating tool. The first dataset was constituted by compounds **1**–**27** for which MIC against several microorganisms were reported by Pinto *et al.* [20]. Since *Microsporum canis* and *Epidermophyton floccosum* demonstrated differential behavior when they were exposed to xanthone treatment, the MIC values against these microorganisms were used in the present research.

**Figure 17:**
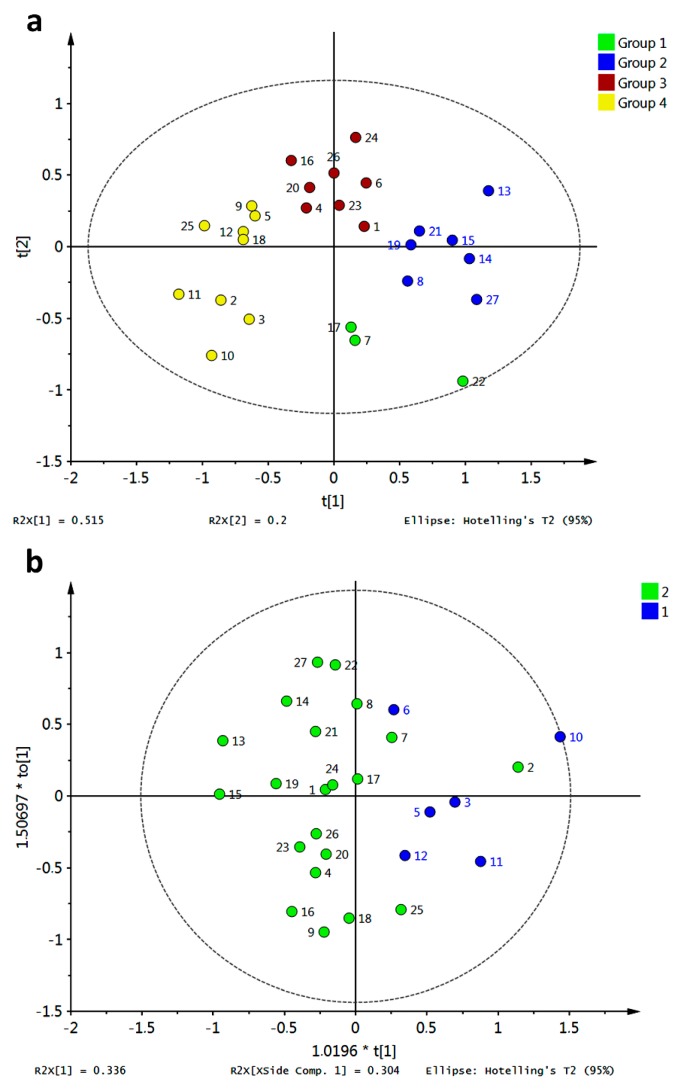

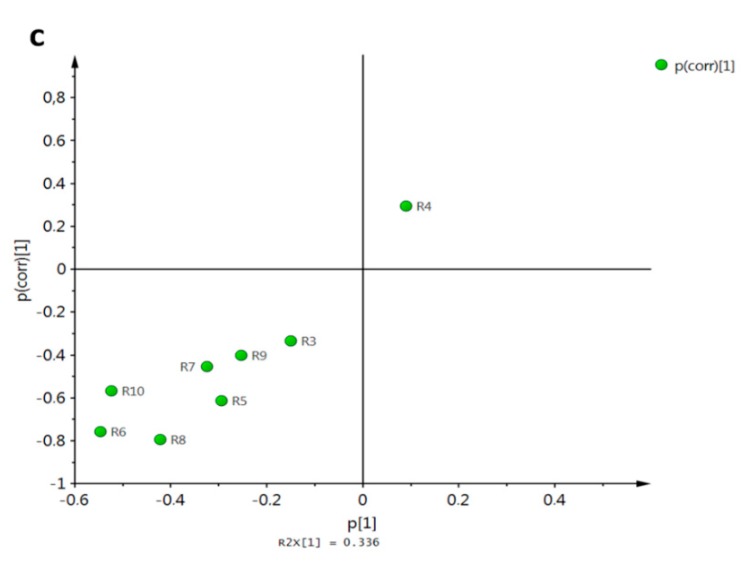
Discrimination of simple xanthones by antifungal activity against *M. canis* and *E. floccosum* based on docking scores (**a**) PCA score plot grouped according to HCA; (**b**) OPLS-DA score plot employing antifungal activity as classification variable (group 1: high to medium activity; group 2: low to absent activity); (**c**) *S*-plot from OPLS-DA.

PCA for the affinity values of compounds **1**–**27** with the tested fungal enzymes (R3–R10) was accomplished and is shown in Figure 17a. Different colors represent different clusters according to HCA. A clear discrimination between the tested xanthones can be observed, allowing us to infer a distinguishing interaction pattern.

Clustering was in good agreement with general structural features as follows: group 3 (in red) consisted of xanthones possessing an OH group at C3 or a OMe group at C1; group 2 (in blue) was characterized mainly by xanthones bearing a OMe at C3; group 4 (in yellow) contained a subgroup defined by xanthones oxygenated at C4 while the another subgroup can be established for xanthones bearing an OH at C2; and group 1 (in green) was constituted by xanthones that did not fulfill the structural requirements of other groups. Partial discrimination of xanthones by activity was achieved with OPLS-DA (Figure 17b). In that analysis, class 1 corresponded to active antifungal xanthones (MIC range of 7.8–31.3 μg/mL [20]). Good correlation between activity and docking scores was observed (highly active xanthones were discriminated along a positive *X*-score). The corresponding *S*-plot (Figure 16c) showed R4 and R6 as main discriminant variables in OPLS-DA: R4 toward a positive *X*-score was (related to active xanthones), while R6 showed the opposite behavior. Therefore, prediction of antifungal activity against *M. canis* and *E. floccosum* could be proposed taking advantage of the affinity energy of the xanthones with the tested enzymes.

**Figure 18:**
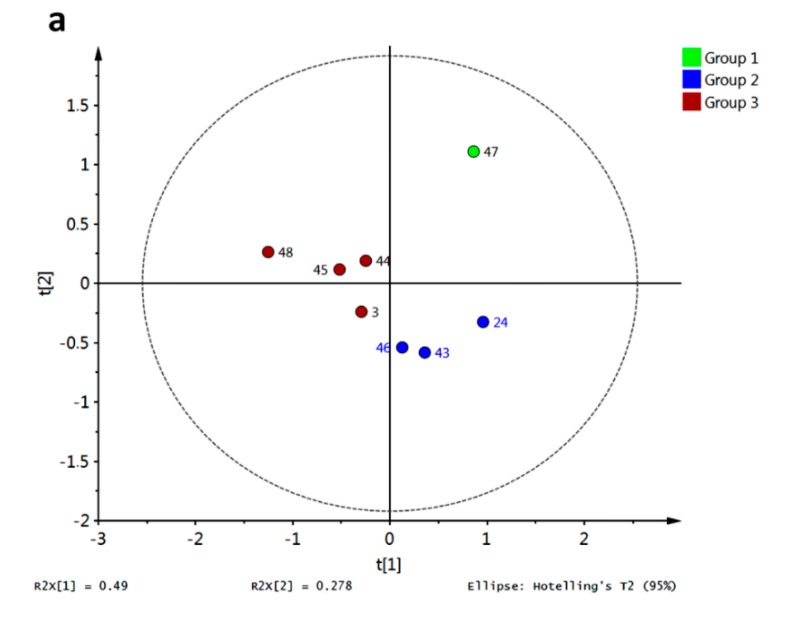

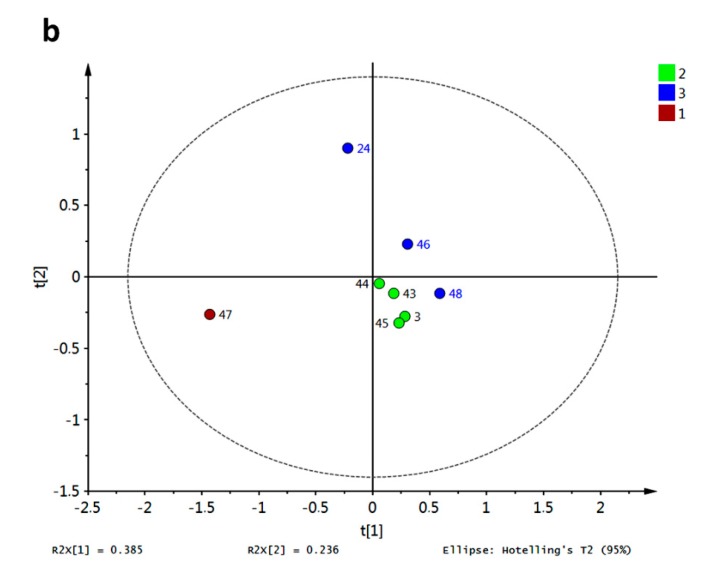
Discrimination of simple xanthones by antifungal activity against *A. flavus* based on docking scores. (**a**) PCA score plot grouped according to HCA; (**b**) PLS-DA score plot employing antifungal activity as classification variable (group 1: highest activity; group 2: medium activity; group 3: lowest activity).

Similar analysis was carried out for compounds **3**, **24**, **43**–**48**, whose antifungal activity was also previously reported [16]. The PCA score plot is shown in Figure 18a. Behavior for compound **47** concerning R3–R10 resulted in a completely different pathway compared with the rest. This xanthone set was only characterized by PeX-type compounds, however no more direct conclusions can be drawn regarding structural dissimilarities per cluster. Discrimination of these xanthones was obtained by PLS-DA with antifungal activity against *Aspergillus flavus* as class observation (Figure 18b). The corresponding score plot showed in red the most active compound (8 μg/mL [16]) while the lowest activity for **24**, **46** and **48** (31 μg/mL [16]) put them far from the rest. Therefore, classification of active xanthones can be achieved by statistical analysis on molecular docking scores being R4, R6 and R10 the most important variables explaining the observed variance.

## 3. Experimental Section

### 3.1. Ligand and Receptor Preparation

A set of 272 xanthones were selected from literature considering those with reported antifungal activity [15,16,17,20] as well as those without previous determined activity [40,41]. Each xanthone was drawn in ChemDraw Ultra (CambridgeSoft, Cambridge, MA, USA) and exported to Spartan’14 (Wavefunction, Inc., Irvine, CA, USA) for conformational searching and subsequent geometry optimization. Conformational searching was carried out by the AM1 semi-empirical method. The lowest energy conformer was subsequently submitted to geometry optimization using the DFT method with the B3LYP functional and 6-31G* as basis set. Each structure was independently saved as a pdb file and transformed then into pdbqt files by the ligand preparation script from MGLTools (The Scripps Research Institute, La Jolla, CA, USA).

Crystal structure data for ribonuclease F1 (Code: 1FUT), cytochrome P450 14 α-sterol demethylase (PDB Code: 1EA1), α-l-arabinofuranosidase (PDB Code: 1QW9), α-fucosidase (PDB Code: 1ODU), nitric oxide reductase (PDB Code: 3AYG), *N*-myristoyltransferase (PDB Code: 1IYK and 1IYL), trichodiene synthase (PDB Code: 1YYR), HIV-1 gp120 (PDB Code: 4DKR) and HIV-1 reverse transcriptase (PDB Code: 2WON) were downloaded from the Protein Data Bank website. Each receptor file was directly prepared employing the plugin for Pymol created by Seeliger and Groot [50]. Binding site definition was determined by comparison with reported interactions for the respective co-crystalized ligand. Flexible residues were selected within 5 Å of this using the plugin for Pymol above mentioned.

### 3.2. Molecular Docking

Molecular docking for the 272 xanthones *versus* the 10 selected receptors was achieved using AutoDock Vina [51]. All calculations were run on an Intel Xeon PC equipped with 32 cores and 64 GB of RAM, running on Ubuntu 12.04. Reproducibility of the calculated affinity energy and the minimum energy pose were evaluated through 10 replicates for each calculation. Affinity energy is reported as mean of the 10 replicates. Differences between the found poses among replicates were analyzed based on RMSD values. Ligand—receptor interactions were visualized and analyzed on Pymol. Selected docked ligand—enzymes complexes were separately saved as pdb file and imported in Discovery Studio (Accelrys Software Inc., San Diego, CA, USA) and LigandScout 2.02 (Inte:Ligand GmbH, Maria Enzersdorf, Austria) to originate the 2D residual interaction diagrams to deeply analyze the binding sites.

### 3.3. Statistical Analysis

Numerical results were compiled and organized as a data matrix containing tested compounds in rows and mean affinity energies in columns. The data matrix was submitted to principal component analysis (PCA), partial least squares-discriminant analysis (PLS-DA) and hierarchical clustering analysis (HCA) using SIMCA 13.0 (Umetrics AB, Umeå, Sweden). Pearson correlations and distribution of the data by boxplots were analyzed and constructed using Matlab R2013a (MathWorks, Natick, MA, USA).

## 4. Conclusions

Highly structurally diverse xanthones were employed in the searching of antifungal and/or antiviral compounds by molecular docking with the aid of statistical analysis. The dataset was assembled by affinity scores of 272 compounds towards 10 enzymes involved in vital metabolic processes of microorganisms. Medium to excellent affinity values were found for the vast majority of the tested xanthones regarding the corresponding co-crystallized inhibitors, which were used as controls. Due to the great structural diversity, no direct pharmacophore information can be extracted, but classification of xanthones according to the presence or absence of prenyl groups or its cyclic biosynthetically related derivatives was found. In general, prenylated xanthones were able to establish significantly stronger complexes with the tested enzymes. Calculated affinities were not related among enzymes, indicating completely different interaction pathways. Moreover, affinity–activity relationships for simple xanthones were interestingly established. From this fact, the calculated interaction with enzymes R4 and R10 resulted to be the most important for discrimination of markedly active xanthones. Differentiation of xanthones based on the relation between antifungal activity and docking affinity let to conclude the importance of this enzymatic set in antifungal drug discovery. For its part, some compounds were defined as potential inhibitors for the analyzed enzymes, including **215** and **223** as potent probable inhibitors of HIV-1 reverse transcriptase. Antiviral and antifungal agents based on xanthones have important advantages such as the availability as natural compounds or the possibility to be synthesized easily and the potential to interact with some important targets in microorganisms. Xanthones could therefore serve as a novel generation of antimicrobial agents as a strategy for responding to outbreaks of resistant viral and fungal infections. However, further QSAR and *in vitro* studies should be performed to validate the *in-silico* data.

## Supplementary Materials

Supplementary materials can be accessed at: http://www.mdpi.com/1420-3049/20/07/13165/s1.
