# Supplementary material for: Molecular Docking and Multivariate Analysis of Xanthones as Antimicrobial and Antiviral Agents
Source: Molecules. 2015 Jul 21;20(7):13165–204. doi: 10.3390/molecules200713165 (PMC6332503; doi:10.3390/molecules200713165)
Supplement: Supplementary file 1 [file molecules-20-13165-s001.pdf]

Supplementary Materials

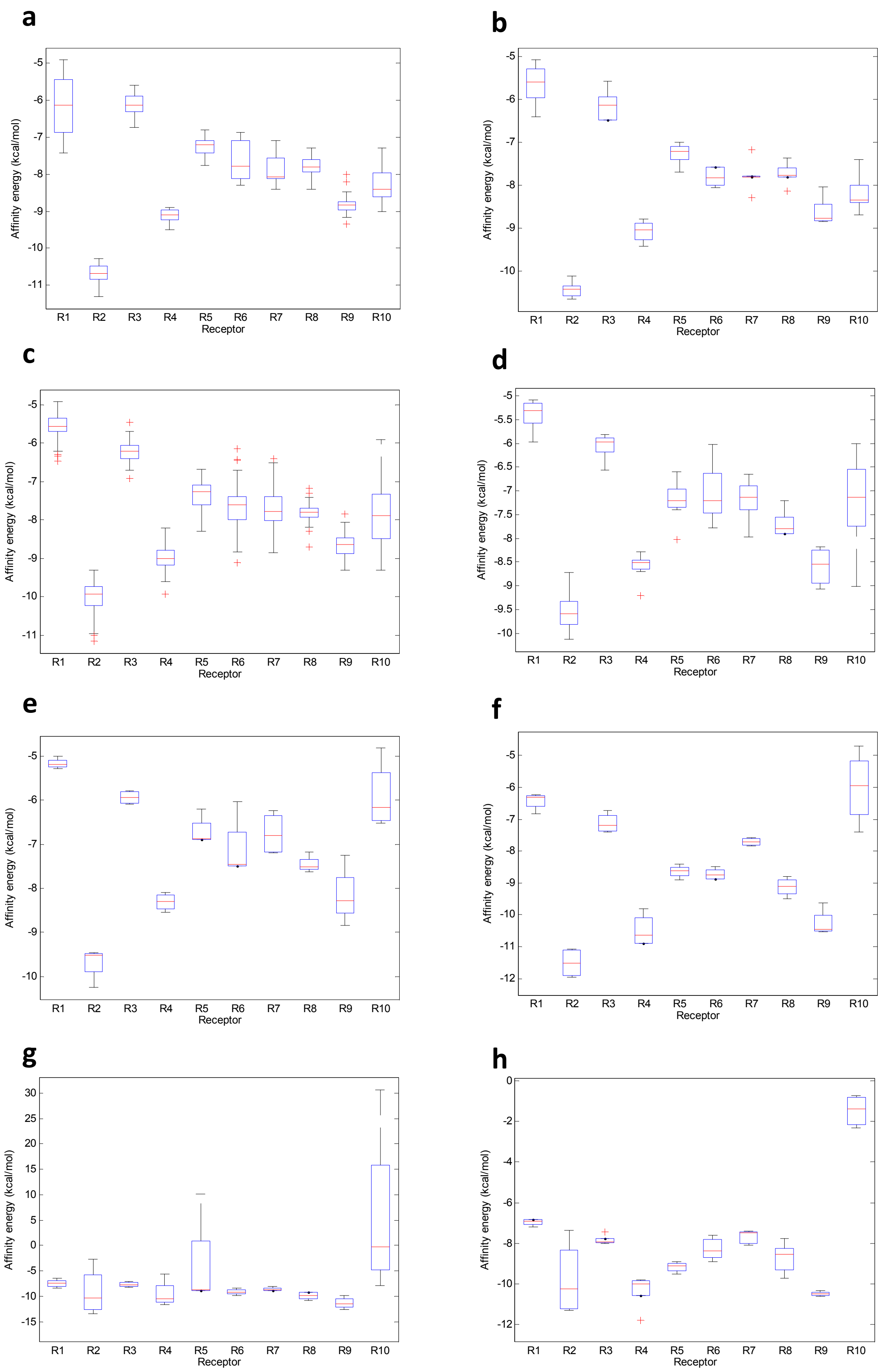

Figure S1. Boxplot of affinity energy values datasets for R1-R10. (**a**) DX; (**b**) TrX; (**c**) TeX; (**d**) PeX; (**e**) HX; (**f**) PePX; (**g**) Dim; (**h**) XD.

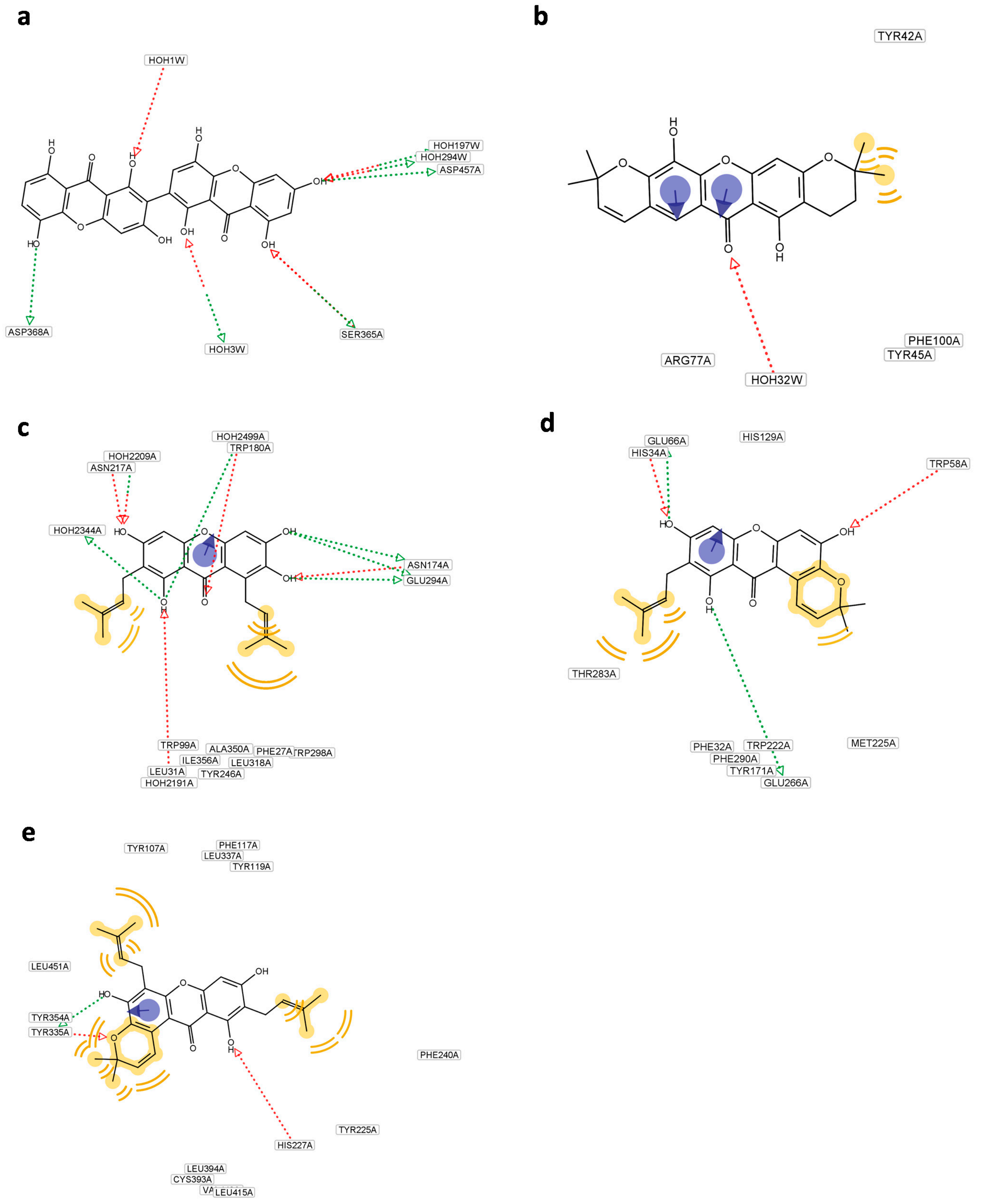

Figure S2. Residual interaction maps for some xanthones. (**a**) **266**—R1; (**b**) **214**—R3; (**c**) **166**—R5; (**d**) **225**—R6; (**e**) **256**—R9.

Table S1. Systematic name of the tested xanthones.

ID	Name	ID	Name
**1**	9*H*-xanthen-9-one	**29**	3,5-dimethoxy-9*H*-xanthen-9-one
**2**	1-hydroxy-9*H*-xanthen-9-one	**30**	1,3-dimethoxy-9*H*-xanthen-9-one
**3**	2-hydroxy-9*H*-xanthen-9-one	**31**	2,7-dimethoxy-9*H*-xanthen-9-one
**4**	3-hydroxy-9*H*-xanthen-9-one	**32**	2-hydroxy-1,8-dimethoxy-9*H*-xanthen-9-one
**5**	4-hydroxy-9*H*-xanthen-9-one	**33**	1,2,7-trimethoxy-9*H*-xanthen-9-one
**6**	4-hydroxy-9*H*-xanthen-9-one	**34**	1,5-dihydroxy-3-methoxy-9*H*-xanthen-9-one
**7**	1-methoxy-9*H*-xanthen-9-one	**35**	1,6-dihydroxy-5-methoxy-9*H*-xanthen-9-one
**8**	2-methoxy-9*H*-xanthen-9-one	**36**	1-hydroxy-5,6-dimethoxy-9*H*-xanthen-9-one
**9**	3-methoxy-9*H*-xanthen-9-one	**37**	1,5-dihydroxy-6-methoxy-9*H*-xanthen-9-one
**10**	4-methoxy-9*H*-xanthen-9-one	**38**	1-hydroxy-2,3,5-trimethoxy-9*H*-xanthen-9-one
**11**	1,2-dihydroxy-9*H*-xanthen-9-one	**39**	1-hydroxy-2,3,7-trimethoxy-9*H*-xanthen-9-one
**12**	2,3-dihydroxy-9*H*-xanthen-9-one	**40**	1-hydroxy-3,4,5-trimethoxy-9*H*-xanthen-9-one
**13**	3,4-dihydroxy-9*H*-xanthen-9-one	**41**	1-hydroxy-3,4,7-trimethoxy-9*H*-xanthen-9-one
**14**	3,5-dihydroxy-9*H*-xanthen-9-one	**42**	1-hydroxy-3,5,6-trimethoxy-9*H*-xanthen-9-one
**15**	3,6-dihydroxy-9*H*-xanthen-9-one	**43**	1-hydroxy-3,5,8-trimethoxy-9*H*-xanthen-9-one
**16**	1,7-dihydroxy-9*H*-xanthen-9-one	**44**	1-hydroxy-3,6,7-trimethoxy-9*H*-xanthen-9-one
**17**	1,5-dihydroxy-9*H*-xanthen-9-one	**45**	1-hydroxy-3,6,8-trimethoxy-9*H*-xanthen-9-one
**18**	3-hydroxy-4-methoxy-9*H*-xanthen-9-one	**46**	8-hydroxy-1,2,6-trimethoxy-9*H*-xanthen-9-one
**19**	4-hydroxy-3-methoxy-9*H*-xanthen-9-one	**47**	2-hydroxy-1,3,7-trimethoxy-9*H*-xanthen-9-one
**20**	3-hydroxy-5-methoxy-9*H*-xanthen-9-one	**48**	7-hydroxy-2,3,4-trimethoxy-9*H*-xanthen-9-one
**21**	2-hydroxy-1-methoxy-9*H*-xanthen-9-one	**49**	3-hydroxy-1,2,4-trimethoxy-9*H*-xanthen-9-one
**22**	3-hydroxy-2-methoxy-9*H*-xanthen-9-one	**50**	3-hydroxy-1,2,7-trimethoxy-9*H*-xanthen-9-one
**23**	1-hydroxy-5-methoxy-9*H*-xanthen-9-one	**51**	3-hydroxy-1,5,6-trimethoxy-9*H*-xanthen-9-one
**24**	5-hydroxy-1-methoxy-9*H*-xanthen-9-one	**52**	6-hydroxy-1,2,8-trimethoxy-9*H*-xanthen-9-one
**25**	1-hydroxy-7-methoxy-9*H*-xanthen-9-one	**53**	4-hydroxy-2,3,6-trimethoxy-9*H*-xanthen-9-one
**26**	1,2-dimethoxy-9*H*-xanthen-9-one	**54**	5-hydroxy-1,2,3-trimethoxy-9*H*-xanthen-9-one
**27**	2,3-dimethoxy-9*H*-xanthen-9-one	**55**	6-hydroxy-1,3,5-trimethoxy-9*H*-xanthen-9-one
**28**	3,4-dimethoxy-9*H*-xanthen-9-one	**56**	7-hydroxy-1,2,3-trimethoxy-9*H*-xanthen-9-one

Table S1. *Cont.*

ID	Name	ID	Name
**57**	2-hydroxy-1,6,8-trimethoxy-9*H*-xanthen-9-one	**85**	2,8-dihydroxy-1,6-dimethoxy-9*H*-xanthen-9-one
**58**	8-hydroxy-1,3,5-trimethoxy-9*H*-xanthen-9-one	**86**	1,8-dihydroxy-2,7-dimethoxy-9*H*-xanthen-9-one
**59**	1,3,5-trihydroxy-2-methoxy-9*H*-xanthen-9-one	**87**	1,8-dihydroxy-3,5-dimethoxy-9*H*-xanthen-9-one
**60**	1,2-dihydroxy-5,6-dimethoxy-9*H*-xanthen-9-one	**88**	1,8-dihydroxy-3,6-dimethoxy-9*H*-xanthen-9-one
**61**	1,3-dihydroxy-2,5-dimethoxy-9*H*-xanthen-9-one	**89**	1,8-dihydroxy-2,6-dimethoxy-9*H*-xanthen-9-one
**62**	1,3-dihydroxy-2,7-dimethoxy-9*H*-xanthen-9-one	**90**	2,4-dihydroxy-3,6-dimethoxy-9*H*-xanthen-9-one
**63**	1,3-dihydroxy-2,8-dimethoxy-9*H*-xanthen-9-one	**91**	2,5-dihydroxy-1,6-dimethoxy-9*H*-xanthen-9-one
**64**	1,3-dihydroxy-4,5-dimethoxy-9*H*-xanthen-9-one	**92**	2,6-dihydroxy-1,5-dimethoxy-9*H*-xanthen-9-one
**65**	1,3-dihydroxy-4,7-dimethoxy-9*H*-xanthen-9-one	**93**	2,7-dihydroxy-1,8-dimethoxy-9*H*-xanthen-9-one
**66**	1,3-dihydroxy-5,6-dimethoxy-9*H*-xanthen-9-one	**94**	3,8-dihydroxy-1,2-dimethoxy-9*H*-xanthen-9-one
**67**	1,3-dihydroxy-5,8-dimethoxy-9*H*-xanthen-9-one	**95**	2,6-dihydroxy-1,8-dimethoxy-9*H*-xanthen-9-one
**68**	1,3-dihydroxy-6,7-dimethoxy-9*H*-xanthen-9-one	**96**	3,6-dihydroxy-1,5-dimethoxy-9*H*-xanthen-9-one
**69**	6,8-dihydroxy-1,2-dimethoxy-9*H*-xanthen-9-one	**97**	1,6-dihydroxy-2,8-dimethoxy-9*H*-xanthen-9-one
**70**	1,4-dihydroxy-3,5-dimethoxy-9*H*-xanthen-9-one	**98**	4,6-dihydroxy-1,3-dimethoxy-9*H*-xanthen-9-one
**71**	1,5-dihydroxy-2,3-dimethoxy-9*H*-xanthen-9-one	**99**	5,6-dihydroxy-1,3-dimethoxy-9*H*-xanthen-9-one
**72**	1,5-dihydroxy-3,4-dimethoxy-9*H*-xanthen-9-one	**100**	1,2,3-trihydroxy-5-methoxy-9*H*-xanthen-9-one
**73**	1,5-dihydroxy-3,7-dimethoxy-9*H*-xanthen-9-one	**101**	1,2,8-trihydroxy-3-methoxy-9*H*-xanthen-9-one
**74**	1,5-dihydroxy-3,8-dimethoxy-9*H*-xanthen-9-one	**102**	1,3,5-trihydroxy-2-methoxy-9*H*-xanthen-9-one
**75**	1,5-dihydroxy-6,7-dimethoxy-9*H*-xanthen-9-one	**103**	1,3,5-trihydroxy-6-methoxy-9*H*-xanthen-9-one
**76**	1,6-dihydroxy-3,5-dimethoxy-9*H*-xanthen-9-one	**104**	1,3,6-trihydroxy-5-methoxy-9*H*-xanthen-9-one
**77**	1,6-dihydroxy-3,7-dimethoxy-9*H*-xanthen-9-one	**105**	1,3,6-trihydroxy-7-methoxy-9*H*-xanthen-9-one
**78**	1,6-dihydroxy-5,7-dimethoxy-9*H*-xanthen-9-one	**106**	1,3,7-trihydroxy-6-methoxy-9*H*-xanthen-9-one
**79**	3,8-dihydroxy-1,2-dimethoxy-9*H*-xanthen-9-one	**107**	2,6,8-trihydroxy-1-methoxy-9*H*-xanthen-9-one
**80**	4,10-dihydroxy-11*H*-[1,3]dioxolo[4,5-*a*]xanthen-11-one	**108**	1,3,8-trihydroxy-5-methoxy-9*H*-xanthen-9-one
**81**	1,7-dihydroxy-2,3-dimethoxy-9*H*-xanthen-9-one	**109**	1,6,8-trihydroxy-2-methoxy-9*H*-xanthen-9-one
**82**	1,7-dihydroxy-3,4-dimethoxy-9*H*-xanthen-9-one	**110**	1,4,7-trihydroxy-3-methoxy-9*H*-xanthen-9-one
**83**	1,7-dihydroxy-3,5-dimethoxy-9*H*-xanthen-9-one	**111**	2,5,8-trihydroxy-1-methoxy-9*H*-xanthen-9-one
**84**	1,7-dihydroxy-3,6-dimethoxy-9*H*-xanthen-9-one	**112**	1,4,8-trihydroxy-3-methoxy-9*H*-xanthen-9-one

Table S1. *Cont.*

ID	Name	ID	Name
**113**	1,5,8-trihydroxy-3-methoxy-9*H*-xanthen-9-one	**141**	2,8-dihydroxy-1,5,6-trimethoxy-9*H*-xanthen-9-one
**114**	1,5,6-trihydroxy-3-methoxy-9*H*-xanthen-9-one	**142**	1,3,8-trihydroxy-4,5-dimethoxy-9*H*-xanthen-9-one
**115**	1,5,7-trihydroxy-3-methoxy-9*H*-xanthen-9-one	**143**	3,6,8-trihydroxy-1,2-dimethoxy-9*H*-xanthen-9-one
**116**	1,6,7-trihydroxy-3-methoxy-9*H*-xanthen-9-one	**144**	8-hydroxy-1,2,3,4,6-pentamethoxy-9*H*-xanthen-9-one
**117**	1,6,8-trihydroxy-2-methoxy-9*H*-xanthen-9-one	**145**	1,8-dihydroxy-2,3,4,6-tetramethoxy-9*H*-xanthen-9-one
**118**	1,2,8-trihydroxy-6-methoxy-9*H*-xanthen-9-one	**146**	1,8-dihydroxy-2,3,4,6-tetramethoxy-9*H*-xanthen-9-one
**119**	1,2,8-trihydroxy-3-methoxy-9*H*-xanthen-9-one	**147**	1,2,3,4,6,7-hexamethoxy-9*H*-xanthen-9-one
**120**	2,4,5-trihydroxy-1-methoxy-9*H*-xanthen-9-one	**148**	1,3,5,6-tetrahydroxy-2-(3-methylbut-2-en-1-yl)-9*H*-xanthen-9-one
**121**	3,5,6-trihydroxy-1-methoxy-9*H*-xanthen-9-one	**149**	1,3,5,6-tetrahydroxy-4-(3-methylbut-2-en-1-yl)-9*H*-xanthen-9-one
**122**	1,3,5,6-tetrahydroxy-9*H*-xanthen-9-one	**150**	1,3-dihydroxy-5,6-dimethoxy-2-(3-methylbut-2-en-1-yl)-9*H*-xanthen-9-one
**123**	1,3,5,7-tetrahydroxy-9*H*-xanthen-9-one	**151**	1,3,6-trihydroxy-5-methoxy-2-(3-methylbut-2-en-1-yl)-9*H*-xanthen-9-one
**124**	1,3,5,8-tetrahydroxy-9*H*-xanthen-9-one	**152**	2,3,6,8-tetrahydroxy-1-(3-methylbut-2-en-1-yl)-9*H*-xanthen-9-one
**125**	1,3,6,7-tetrahydroxy-9*H*-xanthen-9-one	**153**	1,5,8-trihydroxy-3-methoxy-2-(3-methylbut-2-en-1-yl)-9*H*-xanthen-9-one
**126**	1,2,6,8-tetrahydroxy-9*H*-xanthen-9-one	**154**	1,3,5,8-tetrahydroxy-2-(3-methylbut-2-en-1-yl)-9*H*-xanthen-9-one
**127**	8,11-dimethoxy-10*H*-[1,3]dioxolo[4,5-*b*]xanthen-10-one	**155**	1,3,5,8-tetrahydroxy-4-(3-methylbut-2-en-1-yl)-9*H*-xanthen-9-one
**128**	9,11-dimethoxy-10*H*-[1,3]dioxolo[4,5-*b*]xanthen-10-one	**156**	1,4,6-trihydroxy-5-methoxy-7-(3-methylbut-2-en-1-yl)-9*H*-xanthen-9-one
**129**	1,2,3,7-tetramethoxy-9*H*-xanthen-9-one	**157**	3,4,8-trihydroxy-2-methoxy-1-(3-methylbut-2-en-1-yl)-9*H*-xanthen-9-one
**130**	1,2,6,8-tetramethoxy-9*H*-xanthen-9-one	**158**	1,4,5,6-tetrahydroxy-2-(2-methylbut-3-en-2-yl)-9*H*-xanthen-9-one
**131**	1,3,4,5-tetramethoxy-9*H*-xanthen-9-one	**159**	1,5,6-trihydroxy-3-methoxy-2-(2-methylbut-3-en-2-yl)-9*H*-xanthen-9-one
**132**	1,3,4,7-tetramethoxy-9*H*-xanthen-9-one	**160**	1,3,6,7-tetrahydroxy-4-(2-methylbut-3-en-2-yl)-9*H*-xanthen-9-one
**133**	1,3,5,8-tetramethoxy-9*H*-xanthen-9-one	**161**	1,2,5,6-tetrahydroxy-4-(2-methylbut-3-en-2-yl)-9*H*-xanthen-9-one
**134**	1,3,6,7-tetramethoxy-9*H*-xanthen-9-one	**162**	2,5,6-trihydroxy-1-methoxy-4-(2-methylbut-3-en-2-yl)-9*H*-xanthen-9-one
**135**	2,3,4,5-tetramethoxy-9*H*-xanthen-9-one	**163**	1,3,5,6-tetrahydroxy-2-(3-hydroxy-3-methylbutyl)-9*H*-xanthen-9-one
**136**	1-hydroxy-2,3,4,5-tetramethoxy-9*H*-xanthen-9-one	**164**	1,3,6-trihydroxy-7-methoxy-2,5-bis(3-methylbut-2-en-1-yl)-9*H*-xanthen-9-one
**137**	1-hydroxy-2,3,4,7-tetramethoxy-9*H*-xanthen-9-one	**165**	1,3,6-trihydroxy-5-methoxy-2,7-bis(3-methylbut-2-en-1-yl)-9*H*-xanthen-9-one
**138**	8-hydroxy-1,2,5,6-tetramethoxy-9*H*-xanthen-9-one	**166**	1,3,6,7-tetrahydroxy-2,8-bis(3-methylbut-2-en-1-yl)-9*H*-xanthen-9-one
**139**	1-hydroxy-3,5,6,7-tetramethoxy-9*H*-xanthen-9-one	**167**	1,6-dihydroxy-3,7-dimethoxy-2,8-bis(3-methylbut-2-en-1-yl)-9*H*-xanthen-9-one
**140**	8-hydroxy-1,2,4,6-tetramethoxy-9*H*-xanthen-9-one	**168**	1,3,6-trihydroxy-7-methoxy-2,8-bis(3-methylbut-2-en-1-yl)-9*H*-xanthen-9-one

Table S1. *Cont.*

ID	Name	ID	Name
**169**	1,3,5,6-tetrahydroxy-2,4-bis(3-methylbut-2-en-1-yl)-9*H*-xanthen-9-one	**183**	1,3,6-trihydroxy-8-(3-hydroxy-3-methylbutyl)-7-methoxy-2-(3-methylbut-2-en-1-yl)-9*H*-xanthen-9-one
**170**	1,3,5,6-tetrahydroxy-2,7-bis(3-methylbut-2-en-1-yl)-9*H*-xanthen-9-one	**184**	1,3,6-trihydroxy-2-(3-hydroxy-3-methylbutyl)-7-methoxy-8-(3-methylbut-2-en-1-yl)-9*H*-xanthen-9-one
**171**	1,3,5,8-tetrahydroxy-2,4-bis(3-methylbut-2-en-1-yl)-9*H*-xanthen-9-one	**185**	1,3,6,7-tetrahydroxy-8-(3-hydroxy-3-methylbutyl)-2-(3-methylbut-2-en-1-yl)-9*H*-xanthen-9-one
**172**	1,3,5,8-tetrahydroxy-2,4-bis(3-methylbut-2-en-1-yl)-9*H*-xanthen-9-one	**186**	1,3,5,6-tetrahydroxy-2-(3-methylbut-2-en-1-yl)-4-(2-methylbut-3-en-2-yl)-9*H*-xanthen-9-one
**173**	1,6-dihydroxy-3,7-dimethoxy-2,8-bis(3-methylbut-2-en-1-yl)-9*H*-xanthen-9-one	**187**	1,2,5,6-tetrahydroxy-7-(3-methylbut-2-en-1-yl)-4-(2-methylbut-3-en-2-yl)-9*H*-xanthen-9-one
**174**	1,3,6-trihydroxy-7-methoxy-2,8-bis(3-methylbut-2-en-1-yl)-9*H*-xanthen-9-one	**188**	1,3,5,6-tetrahydroxy-7-(3-methylbut-2-en-1-yl)-4-(2-methylbut-3-en-2-yl)-9*H*-xanthen-9-one
**175**	1-hydroxy-3,6,7-trimethoxy-2,8-bis(3-methylbut-2-en-1-yl)-9*H*-xanthen-9-one	**189**	1,3,6,7-tetrahydroxy-5-(3-methylbut-2-en-1-yl)-2-(2-methylbut-3-en-2-yl)-9*H*-xanthen-9-one
**176**	3,6-diethoxy-1-hydroxy-7-methoxy-2,8-bis(3-methylbut-2-en-1-yl)-9*H*-xanthen-9-one	**190**	1,3,5,6-tetrahydroxy-8-(3-methylbut-2-en-1-yl)-2-(2-methylbut-3-en-2-yl)-9*H*-xanthen-9-one
**177**	1-hydroxy-7-methoxy-2,8-bis(3-methylbut-2-en-1-yl)-3,6-dipropoxy-9*H*-xanthen-9-one	**191**	3,6,8-trihydroxy-2-methoxy-1-(3-methylbut-2-en-1-yl)-5-(2-methylbut-3-en-2-yl)-9*H*-xanthen-9-one
**178**	3,6-dibutoxy-1-hydroxy-7-methoxy-2,8-bis(3-methylbut-2-en-1-yl)-9*H*-xanthen-9-one	**192**	1,3,6-trihydroxy-5-methoxy-7-(3-methylbut-2-en-1-yl)-2-(2-methylbut-3-en-2-yl)-9*H*-xanthen-9-one
**179**	1-hydroxy-3,6-diisopropoxy-7-methoxy-2,8-bis(3-methylbut-2-en-1-yl)-9*H*-xanthen-9-one	**193**	1,3,6-trihydroxy-8-(3-hydroxy-3-methylbutyl)-7-methoxy-2-(3-methylbut-2-en-1-yl)-9*H*-xanthen-9-one
**180**	3,6-bis(allyloxy)-1-hydroxy-7-methoxy-2,8-bis(3-methylbut-2-en-1-yl)-9*H*-xanthen-9-one	**194**	2,3,6,8-tetrahydroxy-1,4,7-tris(3-methylbut-2-en-1-yl)-9*H*-xanthen-9-one
**181**	1-hydroxy-7-methoxy-3,6-bis((2-methylallyl)oxy)-2,8-bis(3-methylbut-2-en-1-yl)-9*H*-xanthen-9-one	**195**	3,6,8-trihydroxy-2-methoxy-1,4,7-tris(3-methylbut-2-en-1-yl)-9*H*-xanthen-9-one
**182**	1-hydroxy-7-methoxy-2,8-bis(3-methylbut-2-en-1-yl)-9-oxo-9*H*-xanthene-3,6-diyl diacetate	**196**	3,4,5,8-tetrahydroxy-1,2-bis(3-methylbut-2-en-1-yl)-7-(2-methylbut-3-en-2-yl)-9*H*-xanthen-9-one

Table S1. *Cont.*

ID	Name	ID	Name
**198**	1,3,5,6-tetrahydroxy-4,8-bis(3-methylbut-2-en-1-yl)-2-(2-methylbut-3-en-2-yl)-9*H*-xanthen-9-one	**212**	5,13-dihydroxy-3,3,10,10-tetramethyl-2,3,11,12-tetrahydro-1*H*-dipyrano[3,2-a:2',3'-*i*]xanthen-14(10*H*)-one
**199**	7,12-dihydroxy-9-methoxy-2,2-dimethylpyrano[3,2-*b*]xanthen-6(2*H*)-one	**213**	5,13-dihydroxy-3,3,10,10-tetramethyl-3*H*-dipyrano[3,2-a:2',3'-*i*]xanthen-14(10*H*)-one
**200**	7,9,12-trihydroxy-2,2-dimethylpyrano[3,2-*b*]xanthen-6(2*H*)-one	**214**	7,14-dihydroxy-2,2,10,10-tetramethyl-9,10-dihydro-2*H*-dipyrano[3,2-b:2',3'-*i*]xanthen-6(8*H*)-one
**201**	6,10-dihydroxy-11-methoxy-3,3-dimethylpyrano[2,3-*c*]xanthen-7(3*H*)-one	**215**	5,12-dihydroxy-2,2,10,10-tetramethyl-2*H*-dipyrano[3,2-b:2',3'-*i*]xanthen-6(10*H*)-one
**202**	6,10,11-trihydroxy-3,3-dimethylpyrano[2,3-*c*]xanthen-7(3*H*)-one	**216**	6,13-dihydroxy-3,3,11,11-tetramethyl-3*H*-dipyrano[3,2-b:3',2'-*h*]xanthen-7(11*H*)-one
**203**	6,8,11-trihydroxy-3,3-dimethylpyrano[2,3-*c*]xanthen-7(3*H*)-one	**217**	6,13-dihydroxy-3,3,11,11-tetramethyl-2,3-dihydro-1*H*-dipyrano[3,2-b:3',2'-*h*]xanthen-7(11*H*)-one
**204**	5,8,9-trihydroxy-1,1,2-trimethyl-1H-furo[2,3-*c*]xanthen-6(2*H*)-one	**218**	8,9-dihydroxy-2,3,3,5,6,6-hexamethyl-5,6-dihydro-2*H*-difuro[2,3-a:2',3'-*c*]xanthen-12(3*H*)-one
**205**	5,9-dihydroxy-10-methoxy-1,1,2-trimethyl-1H-furo[2,3-*c*]xanthen-6(2*H*)-one	**219**	5,12-dihydroxy-2,3,3,10,10-pentamethyl-2,3-dihydrofuro[2,3-*h*]pyrano[3,2-*b*]xanthen-6(10*H*)-one
**206**	5,9,10-trihydroxy-1,1,2-trimethyl-1H-furo[2,3-*c*]xanthen-6(2*H*)-one	**220**	5,8-dihydroxy-2,2,10,10-tetramethyl-3,4-dihydro-2*H*-dipyrano[2,3-*a*:2',3'-*i*]xanthen-14(10*H*)-one
**207**	4,8,9-trihydroxy-2,3,3-trimethyl-2H-furo[3,2-*b*]xanthen-5(3*H*)-one	**221**	1,4,7-trihydroxy-2-(2-hydroxypropan-2-yl)-10,10-dimethyl-1,2-dihydrofuro[3,2-*h*]pyrano[3,2-b]xanthen-6(10*H*)-one
**208**	8,9-dihydroxy-4-methoxy-2,3,3-trimethyl-2H-furo[3,2-*b*]xanthen-5(3*H*)-one	**222**	7,9,12-trihydroxy-2,2-dimethyl-10-(2-methylbut-3-en-2-yl)pyrano[3,2-*b*]xanthen-6(2*H*)-one
**209**	9,10-dihydroxy-6-methoxy-3,3-dimethylpyrano[2,3-*c*]xanthen-7(3*H*)-one	**223**	7,9,12-trihydroxy-2,2-dimethyl-8-(2-methylbut-3-en-2-yl)pyrano[3,2-*b*]xanthen-6(2*H*)-one
**210**	5,9,10-trihydroxy-2,2-dimethylpyrano[3,2-*b*]xanthen-6(2*H*)-one	**224**	7,8,12-trihydroxy-2,2-dimethyl-10-(2-methylbut-3-en-2-yl)pyrano[3,2-*b*]xanthen-6(2*H*)-one
**211**	5,9,11-trihydroxy-3,3-dimethylpyrano[3,2-*a*]xanthen-12(3*H*)-one	**225**	5,9,11-trihydroxy-3,3-dimethyl-10-(3-methylbut-2-en-1-yl)pyrano[3,2-*a*]xanthen-12(3*H*)-one

Table S1. *Cont.*

ID	Name	ID	Name
**226**	5,9,11-trihydroxy-3,3-dimethyl-10-(2-methylbut-3-en-2-yl)pyrano[3,2-*a*]xanthen-12(3*H*)-one	**240**	4,8-dihydroxy-9-methoxy-2,3,3-trimethyl-7-(3-methylbut-2-en-1-yl)-2*H*-furo[3,2-*b*]xanthen-5(3*H*)-one
**227**	5,9,11-trihydroxy-3,3-dimethyl-8-(3-methylbut-2-en-1-yl)pyrano[3,2-*a*]xanthen-12(3*H*)-one	**241**	4,8,9-trihydroxy-2,3,3-trimethyl-7-(3-methylbut-2-en-1-yl)-2*H*-furo[3,2-*b*]xanthen-5(3*H*)-one
**228**	5,9,11-trihydroxy-3,3-dimethyl-8-(2-methylbut-3-en-2-yl)pyrano[3,2-*a*]xanthen-12(3*H*)-one	**242**	4,8,9-trihydroxy-2,3,3-trimethyl-11-(3-methylbut-2-en-1-yl)-2*H*-furo[3,2-*b*]xanthen-5(3*H*)-one
**229**	7,10,12-trihydroxy-2,2-dimethyl-8-(2-methylbut-3-en-2-yl)-3,4-dihydropyrano[3,2-*b*]xanthen-6(2*H*)-one	**243**	4,8,9-trihydroxy-2,3,3-trimethyl-11-(2-methylbut-3-en-2-yl)-2*H*-furo[3,2-*b*]xanthen-5(3*H*)-one
**230**	5,9,10-trihydroxy-2,2-dimethyl-12-(2-methylbut-3-en-2-yl)pyrano[3,2-*b*]xanthen-6(2*H*)-one	**244**	4,7,8-trihydroxy-2,3,3-trimethyl-9-(3-methylbut-2-en-1-yl)-2*H*-furo[3,2-*b*]xanthen-5(3*H*)-one
**231**	5,9,10-trihydroxy-2,2-dimethyl-12-(3-methylbut-2-en-1-yl)pyrano[3,2-*b*]xanthen-6(2*H*)-one	**245**	4,6,9-trihydroxy-2-(2-hydroxypropan-2-yl)-11-(3-methylbut-2-en-1-yl)-2*H*-furo[3,2-*b*]xanthen-5(3*H*)-one
**232**	5,9,10-trihydroxy-2,2-dimethyl-8-(3-methylbut-2-en-1-yl)pyrano[3,2-*b*]xanthen-6(2*H*)-one	**246**	5,7,10-trihydroxy-2-(2-hydroxypropan-2-yl)-4-(3-methylbut-2-en-1-yl)-1*H*-furo[2,3-*c*]xanthen-6(2*H*)-one
**233**	5,8-dihydroxy-9-methoxy-2,2-dimethyl-10-(3-methylbut-2-en-1-yl)pyrano[3,2-*b*]xanthen-6(2*H*)-one	**247**	5,9,10-trihydroxy-1,1,2-trimethyl-4-(2-methylbut-3-en-2-yl)-1*H*-furo[2,3-*c*]xanthen-6(2*H*)-one
**234**	5,9-dihydroxy-10-methoxy-2,2-dimethyl-12-(2-methylbut-3-en-2-yl)pyrano[3,2-*b*]xanthen-6(2*H*)-one	**248**	5,9-dihydroxy-10-methoxy-1,1,2-trimethyl-7-(3-methylbut-2-en-1-yl)-1*H*-furo[2,3-*c*]xanthen-6(2*H*)-one
**235**	5,9-dihydroxy-7-(3-hydroxy-3-methylbutyl)-8-methoxy-2,2-dimethyl-3,4-dihydropyrano[3,2-*b*]xanthen-6(2*H*)-one	**249**	5,9-dihydroxy-10-methoxy-2,2-dimethyl-11-(3-methylbut-2-en-1-yl)-3,4-dihydropyrano[2,3-*a*]xanthen-12(2*H*)-one
**236**	5,8,9-trihydroxy-2,2-dimethyl-10-(3-methylbut-2-en-1-yl)pyrano[3,2-*b*]xanthen-6(2*H*)-one	**250**	3,5,9-trihydroxy-10-methoxy-2,2-dimethyl-11-(3-methylbut-2-en-1-yl)-3,4-dihydropyrano[2,3-*a*]xanthen-12(2*H*)-one
**237**	5,9-dihydroxy-8-methoxy-2,2-dimethyl-7-(3-methylbut-2-en-1-yl)pyrano[3,2-*b*]xanthen-6(2*H*)-one	**251**	5,9-dihydroxy-11-(3-hydroxy-3-methylbutyl)-10-methoxy-2,2-dimethyl-3,4-dihydropyrano[2,3-*a*]xanthen-12(2*H*)-one
**238**	5,9-dihydroxy-8-methoxy-2,2-dimethyl-7-(3-methylbut-2-en-1-yl)-3,4-dihydropyrano[3,2-*b*]xanthen-6(2*H*)-one	**252**	9-hydroxy-8,10-dimethoxy-2,2-dimethyl-11-(2-methylbut-3-en-2-yl)pyrano[3,2-*c*]xanthen-7(2*H*)-one
**239**	3,5,9-trihydroxy-8-methoxy-2,2-dimethyl-7-(3-methylbut-2-en-1-yl)-3,4-dihydropyrano[3,2-*b*]xanthen-6(2*H*)-one	**253**	5,12-dihydroxy-1,1,2,10,10-pentamethyl-4-(3-methylbut-2-en-1-yl)-1,2-dihydrofuro[3,2-*h*]pyrano[3,2-*b*]xanthen-6(10*H*)-one

Table S1. *Cont.*

ID	Name	ID	Name
**254**	5,13-dihydroxy-3,3,10,10-tetramethyl-6-(3-methylbut-2-en-1-yl)-3*H*-dipyrano[3,2-a:2',3'-*i*]xanthen-14(10*H*)-one	**267**	(2*S*,3*R*)-4-hydroxy-3-(1-hydroxy-3,6,7-trimethoxy-2,8-bis(3-methylbut-2-en-1-yl)-9-oxo-9*H*-xanthen-4-yl)-2-(2-hydroxypropan-2-yl)-7,8-dimethoxy-6-(3-methylbut-2-en-1-yl)-2*H*-furo[3,2-*b*]xanthen-5(3*H*)-one
**255**	7,9,12-trihydroxy-2,2-dimethyl-8-(3-methylbut-2-en-1-yl)-10-(2-methylbut-3-en-2-yl)pyrano[3,2-*b*]xanthen-6(2*H*)-one	**268**	8-hydroxy-3-(3-hydroxy-4-methoxyphenyl)-2-(hydroxymethyl)-5-methoxy-2*H*-[1,4]dioxino[2,3-*c*]xanthen-7(3*H*)-one
**256**	5,9,11-trihydroxy-3,3-dimethyl-6,10-bis(3-methylbut-2-en-1-yl)pyrano[3,2-*a*]xanthen-12(3*H*)-one	**269**	8-hydroxy-3-(5-hydroxy-2,4-dimethoxyphenyl)-2-(hydroxymethyl)-5-methoxy-2*H*-[1,4]dioxino[2,3-*c*]xanthen-7(3*H*)-one
**257**	5,9,11-trihydroxy-3,3-dimethyl-6,8-bis(3-methylbut-2-en-1-yl)pyrano[3,2-*a*]xanthen-12(3*H*)-one	**270**	8-hydroxy-3-(4-hydroxy-3,5-dimethoxyphenyl)-2-(hydroxymethyl)-5-methoxy-2*H*-[1,4]dioxino[2,3-*c*]xanthen-7(3*H*)-one
**258**	7,10,12-trihydroxy-4-(3-hydroxy-3-methylbutyl)-2,2-dimethyl-8-(2-methylbut-3-en-2-yl)-3,4-dihydropyrano[3,2-*b*]xanthen-6(2*H*)-one	**271**	3-(4-hydroxy-3,5-dimethoxyphenyl)-2-(hydroxymethyl)-5,8-dimethoxy-2*H*-[1,4]dioxino[2,3-*c*]xanthen-7(3*H*)-one
**259**	2,3,5,8-tetrahydroxy-1-(3-hydroxy-3-methylbutyl)-4-(3-methylbut-2-en-1-yl)-7-(2-methylbut-3-en-2-yl)-9*H*-xanthen-9-one	**272**	8,10-dihydroxy-3-(4-hydroxy-3,5-dimethoxyphenyl)-2-(hydroxymethyl)-2*H*-[1,4]dioxino[2,3-*c*]xanthen-7(3*H*)-one
**260**	1,3,5,6-tetrahydroxy-7-methoxy-2,4-bis(3-methylbut-2-en-1-yl)-9*H*-xanthen-9-one		
**261**	5,9,10-trihydroxy-8-methoxy-2,2-dimethylpyrano[3,2-*b*]xanthen-6(2*H*)-one		
**262**	6,9,11-trihydroxy-5-methoxy-3,3-dimethylpyrano[3,2-*a*]xanthen-12(3*H*)-one		
**263**	4,8,9-trihydroxy-11-methoxy-2,3,3-trimethyl-2*H*-furo[3,2-*b*]xanthen-5(3*H*)-one		
**264**	2,2',8,8'-tetrahydroxy-9*H*,9'*H*-[1,1'-bixanthene]-9,9'-dione		
**265**	3-((1,8-dihydroxy-3,6-dimethoxy-9-oxo-9*H*-xanthen-4-yl)oxy)-1,8-dihydroxy-4,6-dimethoxy-9*H*-xanthen-9-one		
**266**	1,1',3,4',5,6',8,8'-octahydroxy-9*H*,9'*H*-[2,2'-bixanthene]-9,9'-dione		
